# RAP1 Protects from Obesity through Its Extratelomeric Role Regulating Gene Expression

**DOI:** 10.1016/j.celrep.2013.05.030

**Published:** 2013-06-20

**Authors:** Paula Martínez, Gonzalo Gómez-López, Fernando García, Evi Mercken, Sarah Mitchell, Juana M. Flores, Rafael de Cabo, Maria A. Blasco

**Affiliations:** 1 Telomeres and Telomerase Group, Molecular Oncology Program; 2 Bioinformatics Core Unit, Structural Biology and Biocomputing Program; 3 Proteomics Core Unit, Biotechnology Program Spanish National Cancer Research Centre (CNIO), Melchor Fernández Almagro 3, Madrid 28029, Spain; 4 Laboratory of Experimental Gerontology, National Institute of Aging, National Institutes of Health, 251 Bayview Boulevard, Baltimore, MD 21224, USA; 5 Animal Surgery and Medicine Department, Faculty of Veterinarian, Complutense University of Madrid, Madrid 28029, Spain

## Abstract

RAP1 is part of shelterin, the protective complex at telomeres. RAP1 also binds along chromosome arms, where it is proposed to regulate gene expression. To investigate the nontelomeric roles of RAP1 in vivo, we generated a RAP1 whole-body knockout mouse. These mice show early onset of obesity, which is more severe in females than in males. *Rap1*-deficient mice show accumulation of abdominal fat, hepatic steatosis, and high-fasting plasma levels of insulin, glucose, cholesterol, and alanine aminotransferase. Gene expression analyses of liver and visceral white fat from *Rap1*-deficient mice before the onset of obesity show deregulation of metabolic programs, including fatty acid, glucose metabolism, and PPAR*α* signaling. We identify *Pparα* and *Pgc1α* as key factors affected by *Rap1* deletion in the liver. We show that RAP1 binds to *Pparα* and *Pgc1α* loci and modulates their transcription. These findings reveal a role for a telomere-binding protein in the regulation of metabolism.

## INTRODUCTION

Mammalian telomeres are composed of tandem repeats of the TTAGGG sequence bound by a specialized protein complex known as shelterin, which protects chromosome ends and regulates telomerase activity (Blasco, 2007; Celli and de Lange, 2005; Chin et al., 1999; d’Adda di Fagagna et al., 2003; de Lange, 2005; Karlseder et al., 1999; Martínez and Blasco, 2010, 2011; Palm and de Lange, 2008; Takai et al., 2003; Tejera et al., 2010; van Steensel et al., 1998). The shelterin complex is composed of six core proteins: TRF1, TRF2, TIN2, POT1, TPP1, and RAP1 (de Lange, 2005). TRF1, TRF2, and POT1 bind directly to telomeric DNA repeats, with TRF1 and TRF2 binding to telomeric double-stranded DNA and POT1 to the 3′ singled-stranded G overhang. TIN2 is able to bind TRF1 and TRF2 through independent domains and to recruit the TPP1-POT1 complex, bridging the different shelterin components (Chen et al., 2008; Kim et al., 2004; Ye et al., 2004). RAP1 binds to telomeric repeats through its interaction with TRF2 (Celli and de Lange, 2005; Li and de Lange, 2003; Li et al., 2000) and protects from telomere fragility and recombination, although it is dispensable for telomere capping (i.e., protection from telomere fusions) (Martinez et al., 2010; Sfeir et al., 2010).

Interestingly, RAP1 is conserved from budding yeast to humans (Li et al., 2000; Shore and Nasmyth, 1987). In budding yeast, scRap1 is the major binding activity at telomeres where it controls telomere length and the establishment of subtelomeric silencing through recruitment of the Sir proteins (Carmen et al., 2002; Hecht et al., 1995; Imai et al., 2000; Kyrion et al., 1993; Marcand et al., 1997; Tanny et al., 1999). Besides its role at telomeres, scRap1 also acts as a transcription factor by controlling the expression of glycolytic enzymes and ribosomal genes, hence its name *R*epressor *a*ctivator *p*rotein 1 (Buchman et al., 1988; Capieaux et al., 1989).

These extratelomeric roles of scRap1 in silencing and in modulating transcription were recently found to be conserved in mammals (Martinez et al., 2010). In particular, by using chromatin immunoprecipitation sequencing (ChIP-seq), we recently demonstrated that mouse RAP1 binds in vivo to telomeric repeats as well as throughout chromosome arms, preferentially by recognition of the (TTAGGG)_2_ consensus motif (Martinez et al., 2010). Nontelomeric RAP1-binding sites are enriched at subtelomeric regions where RAP1 contributes to repression of subtelomeric genes (Kyrion et al., 1993; Martinez et al., 2010; Yang et al., 2009). Interestingly, a significant proportion of the extratelomeric RAP1-binding sites are associated with genes deregulated upon *Rap1* deletion, suggesting a role for RAP1 in transcriptional regulation (Martinez et al., 2010). Intriguingly, gene set enrichment analysis (GSEA) of *Rap1* null mouse embryonic fibroblasts (MEFs) revealed deregulation of pathways involved in cell adhesion and metabolism, including the peroxisome proliferator-activated receptor (PPAR) pathway (Martinez et al., 2010).

Nutrient metabolism and energy homeostasis are tightly controlled by numerous regulatory systems involving specific transcription factors. The PPARs are ligand-activated transcription factors that belong to the superfamily of nuclear hormone receptors and play a key role in nutrient homeostasis (Kersten et al., 2000). Mounting evidence supports a link between the PPARs and diabetes, obesity, dyslipidemia, and inflammation. The PPAR family consists of PPARα, PPARδ (also known as PPARβ), and PPARγ. Ligand-induced activation of PPARs controls the expression of genes involved in energy homeostasis, lipid and lipoprotein metabolism, carbohydrate metabolism, and inflammation (Kidani and Bensinger, 2012). In particular, PPARα is a key regulator of hepatic fatty acid metabolism through direct transcriptional upregulation of genes involved in peroxisomal and mitochondrial β-oxidation pathways, fatty acid uptake, and triglyceride metabolism, especially during fasting (Sanderson et al., 2010). PPARα also has pleiotropic anti-inflammatory and antiproliferative effects. Indeed, synthetic PPARα agonists are used to treat dyslipidemia and to reduce cardiovascular disease and its complications in patients with metabolic syndrome (Lefebvre et al., 2006).

Here, we report that RAP1 plays a role in metabolism through regulation of the PPARα and PGC1α genes. In particular, we show that binding of RAP1 to *Pparα* and *Pgc1α* loci is required for proper *Pparα* and *Pgc1α* transcriptional activation. In the absence of RAP1, PPARα and PGC1α levels are decreased leading to deregulation of several of their target genes and the subsequent deregulation of metabolic pathways involved in energy homeostasis. These molecular defects result in the development of obesity in *Rap1*-deficient mice, which is aggravated with increasing age. Similar to *Pparα*- and *Pgc1α*-deficient mice (Akiyama et al., 2001; Costet et al., 1998; Kim et al., 2003; Lee et al., 1995; Leone et al., 2005), fat accumulation is more pronounced in *Rap1*-deficient females than in males, and they develop pathologies that are reminiscent of metabolic syndrome in humans, further supporting that RAP1 and PPARα are in the same pathway for regulation of metabolism.

## RESULTS

### Generation of Whole-Body *Rap1*-Deficient Mice

To study the nontelomeric roles of RAP1 in the adult organism, we generated a whole-body constitutive *Rap1* knockout mouse, *Rap1*^−/−^, by crossing *Rap1^flox/flox^* mice (Martinez et al., 2010) with transgenic mice carrying the *cre* recombinase under the control of the adenovirus *EIIa* promoter, which targets expression of the *cre* to the early stages of embryonic development, oocytes, and preimplantation embryos (Experimental Procedures). By using this strategy, the resulting gene alterations are genetically fixed and passed onto the progeny (Lakso et al., 1996).

### *Rap1*-Deficient Mice Are Viable but Show an Early Onset of Obesity

*Rap1*-deficient mice were born at the expected Mendelian ratios indicating absence of embryonic lethality. Moreover, *Rap1*-deficient mice showed a normal median survival compared to wild-type controls (Figures S1A–S1C). These findings indicate that RAP1 is dispensable for embryonic development and adult viability, in accordance with normal telomere capping in the absence of Rap1 (Martinez et al., 2010; Sfeir et al., 2010).

**Figure 1:**
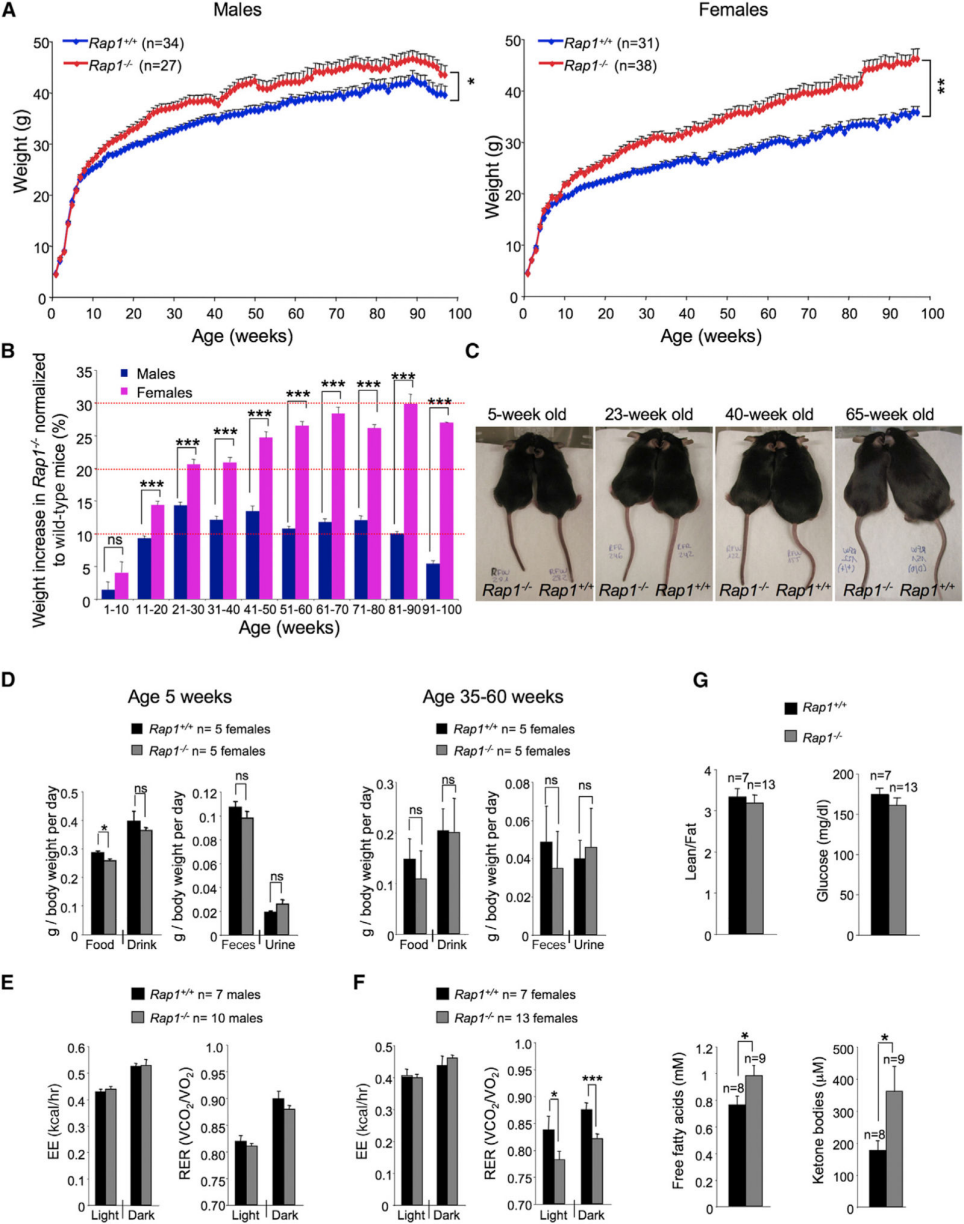
*Rap1* Deficiency Leads to Onset of Obesity (A) Body weight curves of wild-type and *Rap1* null males (left panel) and females (right panel) on a standard chow diet (18% calories from fat). Values and error bars represent the mean and SE, respectively. (B) Weight increment in *Rap1*-deficient mice compared to wild-type controls monitored at 10-week intervals throughout the mice lifespan. (C) Representative images of wild-type and *Rap1*-deficient females at the indicated ages. (D) Relative food intake and output values normalized by body weight monitored during a week period in metabolic cages in young (5 weeks old, left panel) and adult females (35–60 weeks old, right panel). (E and F) EE and RER in male (E) and female (F) mice at the age of 8–12 weeks. (G) Lean/fat ratio, plasma-free fatty acids, ketone bodies, and glucose level in fed state of young females (8–12 weeks old). Error bars in (B) and (D)–(G) represent the SD. Statistical significance was determined by the two-tailed Student’s t test. *p < 0.05; **p < 0.01, ***p < 0.001; ns, not significant. See also Figure S1.

Interestingly, *Rap1*-deficient mice showed a significant increase in the rate of body weight gain compared to wild-type controls under the same feeding conditions (standard mouse chow diet ad libitum; Experimental Procedures) (Figures 1A and 1B). At 10–20 weeks of age, *Rap1*-deficient males showed a 10%–15% increase in body weight compared to wild-type males, and this increased body weight was maintained throughout their lifespan (Figure 1B). This phenotype was more severe in *Rap1*-deficient females, which showed a progressive increase in body weight with time, reaching a 30% increase in body weight compared to wild-type females at 80–90 weeks of age (Figures 1A–1C). The increased body weight of *Rap1*^−/−^ adult mice (35–60 weeks old) cannot be attributed to differences in daily food intake or output (Figure 1D, right panel). Indeed, at a younger age (5 weeks old), *Rap1*^−/−^ females showed a significantly lower food intake compared to wild-type controls (Figure 1D, left panel). Together, these findings indicate that *Rap1* deletion leads to an early onset of obesity, which is more pronounced in females than in males, and cannot be attributed to a higher food intake.

We next determined whether increased body weight in *Rap1*-deficient mice could be due to differences in energy expenditure (EE). To this end, we performed indirect calorimetry analysis in 8-to 12-week-old mice from both genotypes. We found no significant differences between genotypes in EE, oxygen consumption, or locomotor activity, both in males and females (Figures 1E, 1F, S1D, and S1E). Instead, we found a significant lower respiratory exchange rate (RER) in *Rap1*-deficient females compared to wild-type females in both light and dark cycles (Figure 1F). These differences could not be attributed to different body contents because the ratio lean/fat was similar in both groups of females as measured by nuclear magnetic resonance (NMR) (Figure 1G). A lower RER suggests that more fat is being used as energy source.

In order to assess whether there was an effect of *Rap1* deficiency in fatty acid mobilization, we analyzed circulating free fatty acids and ketone bodies, the by-product of fatty acid oxidation (Kersten et al., 1999). Interestingly, we found that young *Rap1*-deficient females show significant higher levels of both free fatty acids and ketone bodies compared to wild-type controls (Figure 1G), in agreement with the lower RER values. This effect, however, was not observed in older females subjected to different types of diets, which were obese (see Figure 4F). Plasma glucose levels were similar between genotypes (Figure 1G).

### *Rap1*-Deficient Mice Accumulate More Fat in Visceral Tissues and Show Signs of Liver Steatosis and Inflammation

**Figure 2:**
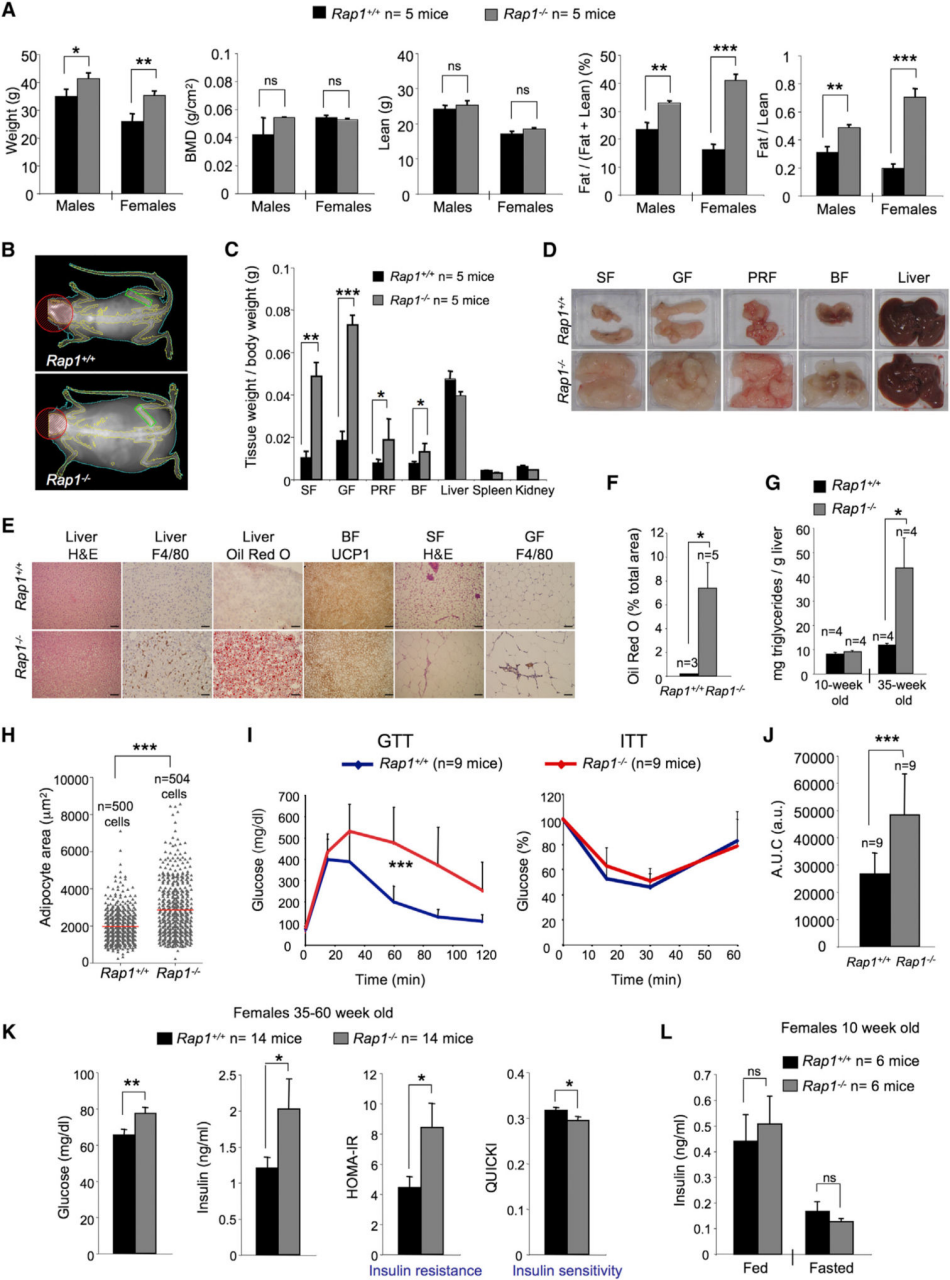
*Rap1*-Deficient Mice Accumulate More Fat and Are Glucose Resistant (A) Body weight, bone mineral density (BMD), lean mass, relative fat mass, and fat/lean ratio in 30-week-old mice of the indicated gender and genotype measured by DXA. (B) Representative DXA images of wild-type and *Rap1*-deficient females. (C) Organ weight-to-total body weight ratios. SF, subcutaneous fat; GF, gonadal fat; PRF, perirenal fat; BF, brown fat. (D) Representative macroscopic images of the indicated tissues and organs. (E) Representative light microscopy images of H&E sections, F4/80 immunohistochemistry, oil red O staining, and UCP1 immunohistochemistry of the indicated tissues (scale bars, 100 µm). (F) Quantification of oil red O-positive areas in liver sections of the indicated genotypes. (G) Quantification of triglyceride content in liver samples of young (10 weeks old) and adult (35 weeks old) females. (H) Quantification of the adipocyte area in abdominal fat depots. (I) GTT and ITT data of nine 40- to 50-week-old wild-type and *Rap1* knockout females. (J) Quantification of the area under the GTT curve (AUC), a.u., arbitrary units. (K) Fasting glucose levels, fasting insulin levels, derived HOMA-IR insulin-resistance quantification, and QUICKI insulin sensitivity quantification of 35- to 60-week-old females. (L) Insulin levels of young females (10 weeks old) during fed and fasted states. Error bars represent SD. Statistical significance was determined by two-tailed Student’s t test. *p < 0.05; **p < 0.01; ***p < 0.001.

To determine the origin of the increased body weight, we performed dual-energy X-ray absorptiometry (DXA), which allows quantification of whole-body fat mass and of the fat-to-lean ratio. We found that *Rap1*^−/−^ mice had a significant relative increase in fat mass at 30 weeks of age in the absence of differences in the bone mass index or in lean mass compared to wild-type controls (Figures 2A and 2B). Fat accumulation was more pronounced in *Rap1*-deficient females than in males (Figures 2A and 2B). In particular, at 30 weeks of age, *Rap1*-deficient males and females presented a 34% and a 40% fat mass relative to lean mass, respectively, compared to 25% and 18% in age-matched wild-type males and females, respectively. Similar results were obtained in older (55 weeks old) mice (data not shown).

We confirmed these findings by determining the relative weight of fat and lean in different tissues. We observed a significant increase in subcutaneous, gonadal, perirenal, and brown fat mass relative to total body weight in *Rap1*-deficient females compared to wild-type controls (Figures 2C and 2D). We did not observe, however, significant differences in the weight of liver, spleen, and kidney (Figures 2C and 2D). Of note, we noticed accumulation of white fat also around brown fat in *Rap1*-deficient mice, which was coincidental with larger intracellular lipid droplets in brown fat tissues (Figures 2D and 2E). In addition, F4/80 staining of liver and white fat sections showed more abundant macrophage infiltrates in *Rap1*-deficient samples compared to the wild-type controls (Figure 2E), indicative of increased inflammation. Hematoxylin and eosin staining (H&E) of white fat and liver sections revealed a larger size of adipocytes and of hepatic lipid deposits, respectively (Figure 2E). Oil red O staining of liver sections confirmed accumulation of large lipid droplets suggestive of liver steatosis (Figure 2E). The oil red O-stained area per section was significantly higher in *Rap1*-deficient livers compared to wild-type controls (Figure 2F). Finally, whereas we found similar amounts of liver triglycerides in young (10 weeks old) females of both genotypes, older *Rap1*-deficient females (35 weeks old) showed a significant 5-fold increase in liver triglycerides compared to wild-type controls, further indicative of liver steatosis (Figure 2G). The adipocyte mean area in abdominal fat depots was also significantly larger in *Rap1*^−/−^ females compared to wild-type controls, indicating that fat accumulation is due to both higher numbers and larger adipocytes (Figure 2H).

### *Rap1*-Deficient Mice Are Glucose Resistant and Show Some Signs of Metabolic Syndrome

To dissect the physiological defects leading to increased body weight in *Rap1*-deficient mice, we tested their ability to respond to glucose and insulin. To this end, we performed glucose and insulin tolerance tests (GTTs and ITTs, respectively) on 35- to 50-week-old females. We found that *Rap1*-deficient females are glucose resistant compared to wild-type controls but show a normal response to exogenously administered insulin (Figure 2I). In particular, the area under the curve (AUC) values for the GTT assays were significantly higher in *Rap1*-deficient females compared with wild-type controls (Figure 2J). Analysis of fasting glucose and insulin levels in a total of 16 mice per genotype at 20–60 weeks of age showed significant higher levels of both glucose and insulin in *Rap1*-deficient mice (Figure 2K). The derived insulin resistance and insulin sensitivity indices, *ho*meostatic *m*odel *a*ssessment (HOMA-IR) and *qu*antitative *i*nsulin sensitivity *c*hec*k i*ndex (QUICKI), respectively, revealed a worsened insulin resistance and decreased insulin sensitivity in *Rap1*-deficient females (Figure 2K). Insulin levels were normal in young 10-week-old females of both genotypes before the onset of obesity, indicating that the glucose-resistance phenotype appears later in life concomitantly with the increased body weight (Figure 2L).

**Figure 3:**
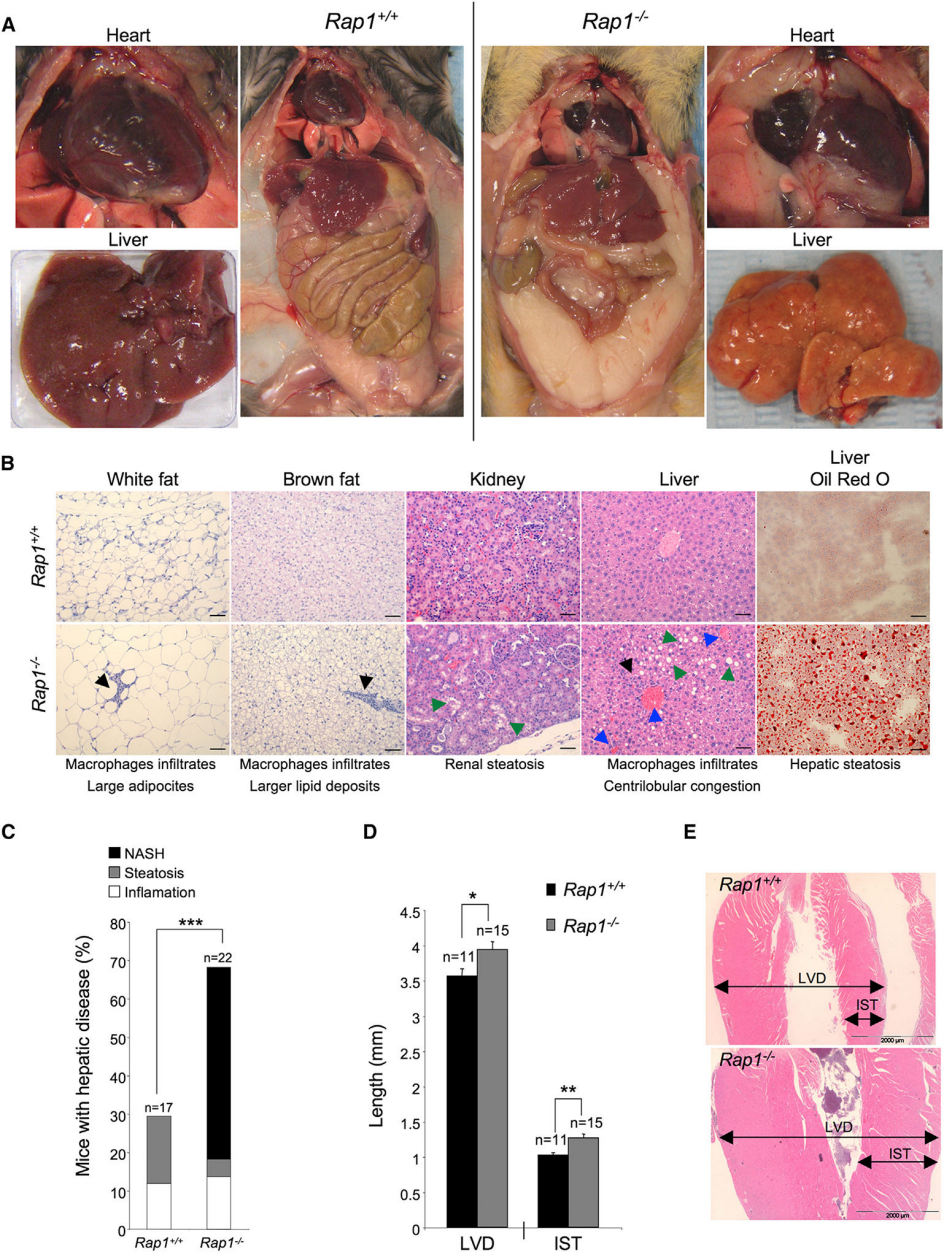
*Rap1*-Deficient Mice Show Signs of Metabolic Syndrome and Cardiopathies at Death (A) Representative images of (left) wild-type and (right) *Rap1* knockout female bodies upon sacrifice at HEP. Mice were 80–90 weeks old. Note the dramatic accumulation of subcutaneous, abdominal, and pericardial fat (magnification images of the heart at the sides) in *Rap1* knockout females. Representative images of liver samples are shown. Notice the fatty liver appearance of *Rap1*-deficient mice. (B) Representative light microscopy images of H&E and red oil O-stained sections of the indicated organs of wild-type and knockout females at death. Note the presence of macrophage infiltrates (black arrowheads) in *Rap1*-deficient white fat, brown fat, and liver compared to wild-type control tissues indicative of inflammatory lesions; note also the larger size of *Rap1*-deficient adipocytes as well as the lipid deposits in brown fat, liver, and kidney (green arrowheads). The liver of *Rap1*-deficient mice presents symptoms of hepatic steatosis and centrilobular vein congestion (blue arrowheads). (C) Incidence of liver disease at time of death in wild-type and *Rap1*-deficient males and females. NASH includes severe steatosis and inflammation, as well as fibrosis in some cases. (D) Quantification of the left ventricular diameter (LVD) and the intraventricular septum thickness (IST). (E) Representative images of wild-type and *Rap1*-deficient heart at death. Error bars represent the SD. Statistical significance was determined by the two-tailed Student’s t test. *p < 0.05; **p < 0.01, ***p < 0.001. See also Figure S2.

In humans, increased body mass (i.e., central obesity), fatty liver, and increased fasting plasma glucose levels are indicative of metabolic syndrome, a condition associated with increased visceral fat, inflammation, and severe cardiovascular problems (Byrne et al., 2009). To address whether *Rap1* deficiency was leading to features of metabolic syndrome in mice, we performed full histopathological analysis of *Rap1*-deficient females at their time of death (Experimental Procedures). We observed large accumulations of subcutaneous and abdominal fat, as well as increased pericardial fat in *Rap1*-deficient females compared to the wild-type controls (Figure 3A). Furthermore, *Rap1*-deficient mice showed macrophage infiltrates in white fat, brown fat, and in the liver, indicative of inflammation (Figure 3B). Lipidosis was also observed in kidneys, although to a lower extent than in the liver (Figure 3B). Hepatic steatosis in *Rap1*-deficient females was manifested by abundant and large lipid deposits in liver sections that in some cases could be readily detected macroscopically by the enlarged size and pale-yellow appearance of the liver (Figures 3A and 3B). Indeed, full histopathological analysis at the time of death revealed that 50% of both male and female *Rap1*-deficient mice showed severe hepatic steatosis and inflammation, a condition that is clinically known as nonalcoholic steatohepatitis (NASH) (Figure 3C). Centrilobular vein congestion was also observed in *Rap1*-deficient livers indicative of cardiopathologies (Figure 3B) (Shibayama, 1987). Indeed, histopathological heart examination revealed increased left ventricular diameter and increased interventricular septum thickness in *Rap1*-deficient mice (Figures 3D and 3E). Of note, we found a similar mouse survival and normal tumor incidence in both genotypes although males showed a trend towards a lower survival (Figure S1A–S1C and S2A). Together, these findings indicate that *Rap1*-deficient mice develop pathologies, some of which are reminiscent of those associated with metabolic syndrome in humans.

Finally, we set out to address whether altered brown adipose tissue (BAT) activity could also contribute to the phenotypes of *Rap1*-deficient mice. BAT plays a role in total energy homeostasis and body weight regulation by dissipating excess energy by the so-called adaptive thermogenesis (Lowell et al., 1993). To this end, we measured glucose uptake in brown fat by PET in a set of adult wild-type and knockout animals. However, no differences in glucose uptake between genotypes could be detected, suggesting the absence of metabolic deregulation in *Rap1*-deficient brown fat (Figures S2B and S2C).

### High-Fat Diet Further Aggravates Obesity and Diabetes in *Rap1*-Deficient Females

**Figure 4:**
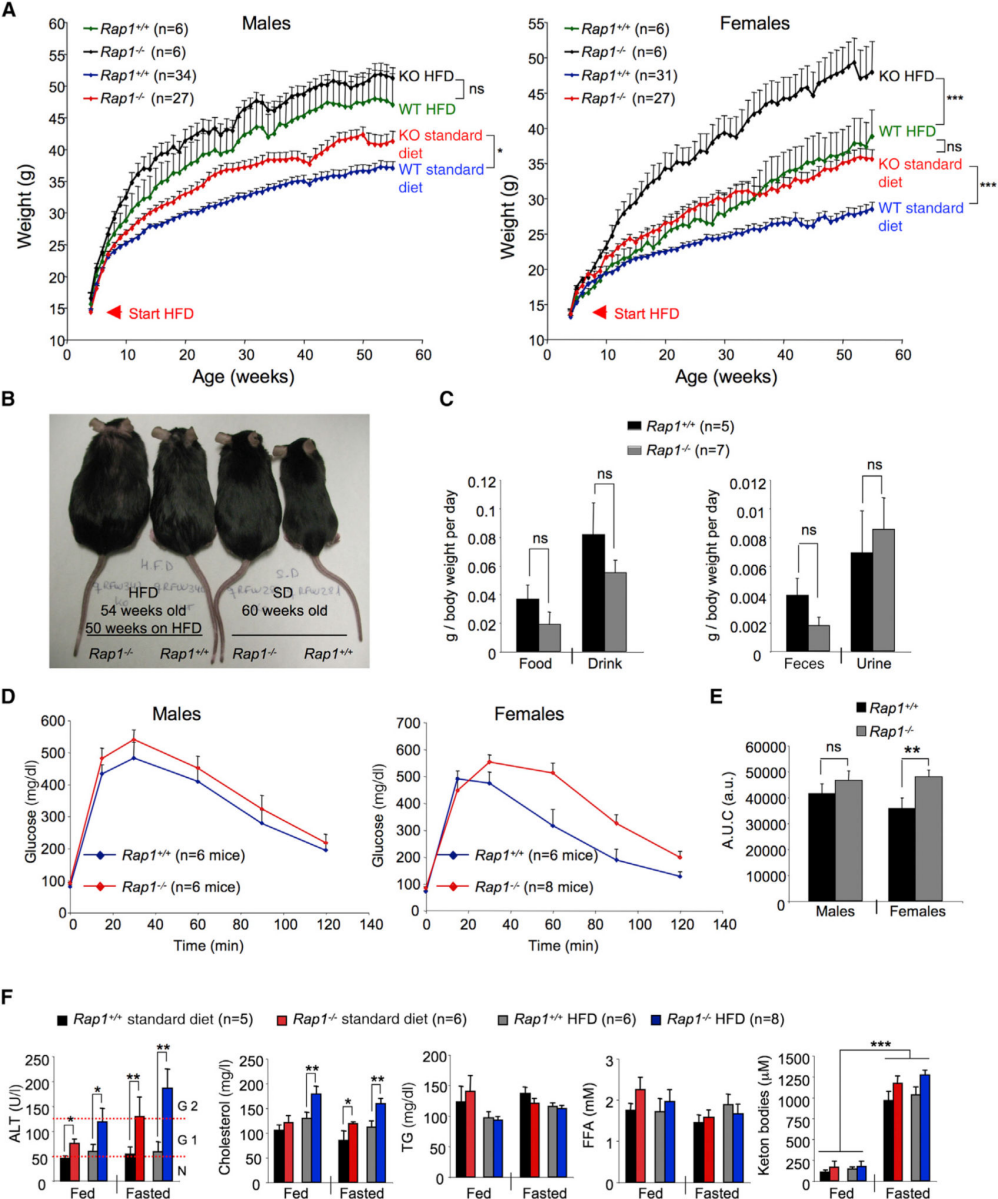
Enhanced Weight Gain and Glucose Resistance in *Rap1* Null Females Subjected to a HFD (A) Weight curves of male and female mice of the indicated phenotypes on a standard diet (18% calories from fat) or HFD (45%calories from fat) commencing at 4 weeks of age. (B) Representative images of female mice of the indicated genotype and age subjected to a HFD during 50 weeks and to a standard diet (“SD”; 60 weeks). (C) Relative food intake and output values normalized by body weight monitored during a week period in metabolic cages in female mice subjected to a standard diet. (D) GTT data of wild-type and *Rap1* knockout male (left) and female (right) mice after 20 weeks on a HFD. (E) Quantification of the area under the GTT curve (AUC). (F) Analysis of plasma parameters in wild-type and *Rap1*-deficient females on a standard diet (75–85 weeks old) and HFD (after 30 weeks on HFD). TG, triglycerides; FFA, free fatty acids. Error bars represent SD. Statistical significance was determined by two-tailed Student’s t test. *p < 0.05; **p < 0.01, ***p < 0.001.

To further understand the origin of obesity and liver steatosis associated with *Rap1* deficiency, we subjected 4-week-old *Rap1^+/+^* and *Rap1*^−/−^ males and females to high-fat diet (HFD) and followed weight gain in a longitudinal manner (weekly measurements) (Experimental Procedures). In males, the HFD resulted in a 10% increase in body weight compared to wild-type controls, although the difference was not significant (Figure 4A). Interestingly, *Rap1*-deficient females showed a faster rate of weight gain compared to wild-type controls from the start of the treatment. In particular, 10 weeks after placement on a HFD, *Rap1*-deficient females gained approximately 35% more weight than wild-type females, and this difference was maintained or increased throughout the treatment (Figures 4A and 4B). In particular, whereas weight increase in wild-type females fed a HFD was approximately 30% higher compared to wild-type mice fed a standard diet, knockout females fed a HFD presented as much as a 70% higher weight gain than wild-type-females fed with a standard diet (Figures 4A and 4B). Interestingly, *Rap1*-deficient females fed with a standard diet gained weight at the same rate as wild-type females fed with a HFD (Figures 4A and 4B), suggesting that the magnitude of the metabolic changes associated with *Rap1* deficiency in female mice is similar to those induced by a HFD. The increased body weight of *Rap1*^−/−^ females fed a HFD could not be attributed to differences in daily food intake or output (Figure 4C).

Next, we performed GTTs and ITTs 20 weeks after placement on a HFD in both genotypes. We found that *Rap1*-deficient females are significantly more glucose resistant than wild-type females on the same diet, whereas no significant differences were observed in males (Figure 4D). In agreement with this, the AUC values revealed a significantly worse glucose tolerance in the *Rap1*-deficient females on a HFD compared to wild-type controls on the same diet (Figure 4E). Upon injection of insulin, HFD-fed animals were almost unresponsive to insulin not being able to remove glucose from blood. However, both male and female wild-type and *Rap1*-deficient mice showed similar ITT curves (data not shown), indicating that HFD affects the response to insulin independently of RAP1.

In order to further understand the metabolic effects of RAP1 abrogation, we analyzed a number of metabolic parameters in the plasma of mice subjected to either a standard diet or a HFD, in fed state or after 16 hr fasting (Figure 4F). We found that *Rap1*-deficient females on both standard diet and HFD showed significantly increased levels of alanine aminotransferase (ALT) compared to wild-type females, indicative of liver dysfunction. Indeed, the ALT levels present in fasted *Rap1*-deficient females on both diets correspond to a grade 2 of hepatotoxicity (126–250 U/l) (Lenaerts et al., 2005). Of note, *Rap1*-deficient females on a standard diet showed higher ALT levels than wild-type controls on a HFD, again indicating the magnitude of the metabolic defects associated with *Rap1* deficiency. Cholesterol levels were also significantly higher in fasted *Rap1*-deficient females compared to wild-type controls on both diets (Figure 4F). In the case of males, we did not find any differences between genotypes, although both ALT and cholesterol levels were significantly elevated in mice on a HFD compared to standard diet (Table S1).

No differences in the levels of free fatty acids, ketone bodies, and triglycerides were found between genotypes (Figure 4F). Similarly, no differences in the levels of lactate, creatinine, total proteins, albumin, urea, phosphorous, and calcium between both genotypes could be detected, indicating no kidney dysfunction (Table S1).

### Metabolic Alterations in *Rap1*-Deficient Tissues Occur in the Absence of Changes in Telomere Length and in the Absence of Telomere Damage

**Figure 5:**
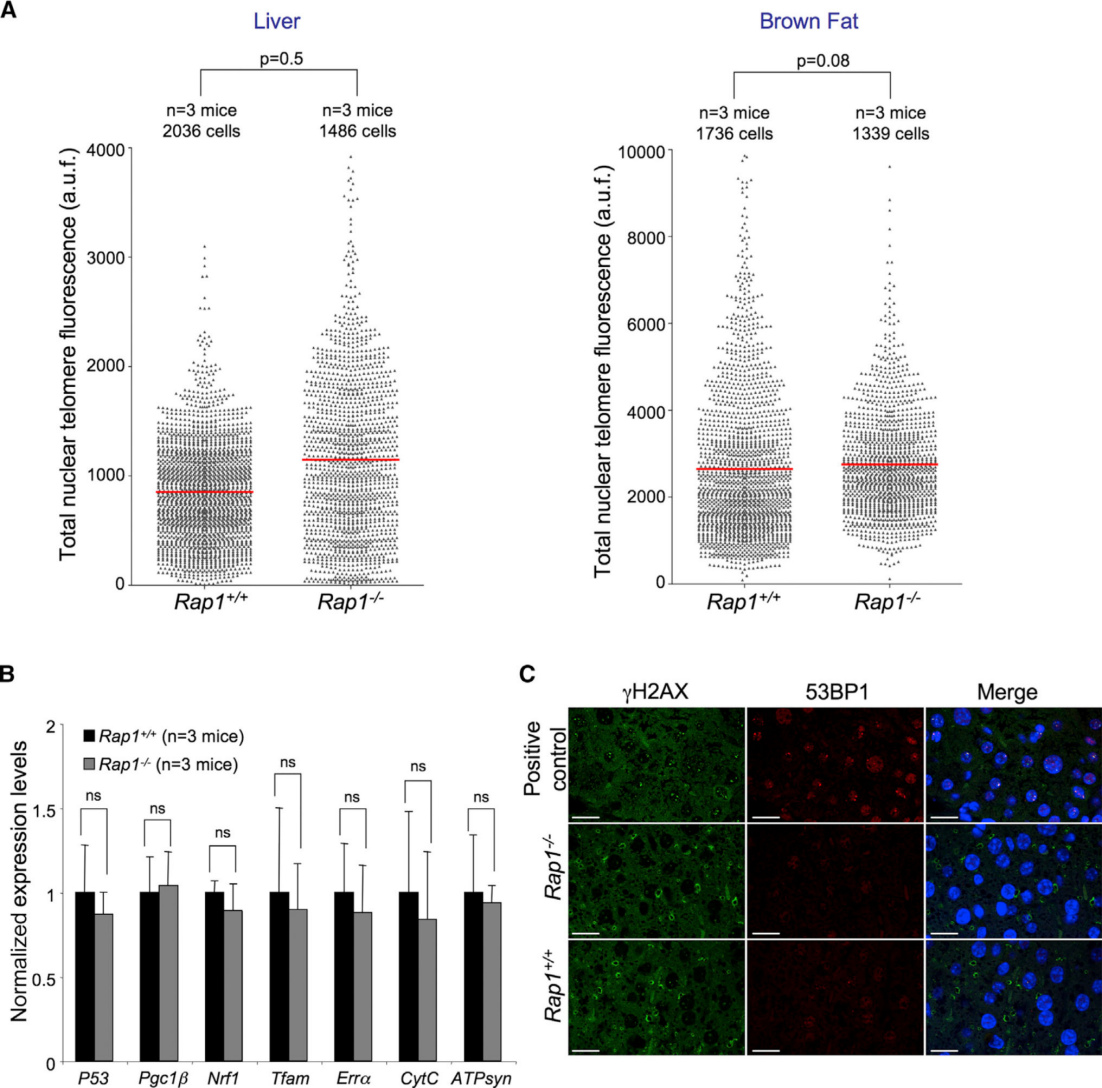
*Rap1* Deficiency Does Not Lead to Changes in Telomere Length in Liver and in Brown Fat (A) Total nuclear telomere fluorescence as determined by Q-FISH on tissue sections. Three 30-week-old females per genotype were used for the analysis. a.u.f., arbitrary units of fluorescence. (B) qPCR validation of OXPHOS genes in liver samples. The results are normalized to wild-type samples. Three independent samples per genotype were analyzed. (C) Representative immunofluorescence images of liver sections from the indicated genotypes stained for γH2AX (green) and 53BP1 (red). Posthepatectomized TRF1-deficient liver sections were used as positive controls for γH2AX and 53BP1. Scale bars, 50 µm. Statistical significance was determined by two-tailed Student’s t test. Error bars represent SD.

Telomere dysfunction owing to extreme telomere shortening is proposed to induce metabolic and mitochondrial compromise (Sahin et al., 2011). In particular, late-generation telomerase-deficient mice show a p53-dependent transcriptional repression of *Pgc1α* and *Pgc1β* and the subsequent downregulation of several of their target genes (*Nrf1, Errα, Tfam*, and *Pparα*), as well as downregulation of members of the oxidative phosphorylation (OXPHOS) pathway (ATP synthase, cytochrome C, and cytochrome C oxidase) (Sahin et al., 2011). In order to address whether the metabolic changes observed in *Rap1*-deficient mice could be the indirect consequence of defects in telomere length homeostasis, we performed quantitative telomere fluorescence in situ hybridization (Q-FISH) analysis on liver and brown fat sections from both genotypes. Telomere fluorescence was similar in liver and brown fat tissues from both genotypes (Figure 5A), indicating that the metabolic changes associated with *Rap1* deficiency are not due to abnormal telomere length.

We next determined the expression levels of *Pgc1β, p53, Nrf1, Errα, Tfam, CytC, and ATPsyn* by qPCR in liver tissue from both genotypes (Figure 5B). In contrast to repression of these genes in late-generation telomerase-deficient mice, we did not find significant changes in their expression in *Rap1*-deficient livers compared to controls (Figure 5B), in line with normal telomere length in *Rap1*-deficient livers. Also in accordance with normal telomere length, we did not find increased telomere damage in liver as indicated by γH2AX and 53BP1stainning (Figure 5C).

### RAP1 Deficiency Affects Metabolic Transcriptional Networks before the Onset of Obesity

We previously reported that RAP1 binds throughout chromosome arms where it is proposed to regulate transcription (Martinez et al., 2010). In order to address whether obesity, diabetes, and other metabolic phenotypes provoked by *Rap1* deficiency could be explained by defined transcriptional changes, we studied gene expression profiles of liver, gonadal white fat, and gastrocnemius muscle from mice from both genotypes. To rule out that the expression changes could be secondary to the obesity phenotype, we performed the gene expression studies before the onset of obesity. To this end, we used young females (10 weeks old) with a similar mean body weight of around 19 g in both genotypes. GSEA of liver showed alterations in many metabolic pathways (Figure S3A; Table S2). In particular, fatty acid metabolism, androgen and estrogen metabolism, biosynthesis of steroids, pyruvate metabolism, and PPAR signaling pathway were significantly deregulated in the liver of *Rap1*-deficient females. In gonadal white fat, *Rap1*-deficient mice showed an enrichment of inflammation/immunity and cell adhesion/cell-cell interaction networks (Figure S3B; Table S3). Similarly, metabolic pathways, such as OXPHOS and PPAR signaling pathways, also showed an enriched signature in wild-type gonadal fat (Figure S3B; Table S3). In contrast, GSEA of muscle did not render any significantly deregulated pathway, suggesting that the RAP1-mediated metabolic phenotype does not stem from transcriptional deregulation in muscle (data not shown). In agreement with this notion, we did not find differences in the abundance of type I (high oxidative potential) and type II (low oxidative potential) fibers in the gastrocnemius muscle (Lin et al., 2002). We also found a similar succinate dehydrogenase (SDH) staining in the gastrocnemius of young females (8 weeks old) from both genotypes (Figures S2D and S2E), thus indicating that metabolic phenotypes associated with *Rap1* deficiency are not mediated by the muscle.

We further confirmed deregulation of key metabolic pathways in older females at the onset of obesity by using both transcriptome and proteome analyses (Experimental Procedures). In particular, we studied wild-type and *Rap1*-deficient females at 34 weeks of age, with mean body weights of 24 and 36 g, respectively. Differential gene expression analysis revealed that 671 probes deregulated, corresponding to 618 genes (false discovery rate [FDR] <0.15) in *Rap1*-deficient liver (Table S4). Gene Ontology (GO) analysis of the results showed a significant upregulation of genes involved in different metabolic pathways including the organic acid, lipid, fatty acid, steroid, cholesterol, and carboxylic acid metabolism in *Rap1*-deficient livers (Table S5). In addition, GSEA of *Rap1*-deficient livers showed alteration of many metabolic pathways, including the branched chain amino acid degradation, the PPAR signaling pathway, glycerolipids, and fatty acid metabolism, which showed a highly significant enrichment in *Rap1*-deficient livers (FDR <0.01), reflecting alterations in lipid homeostasis. Several routes within carbohydrate metabolism such as glycolysis and gluconeogenesis as well as diabetes pathways were also found deregulated (Table S6). Of interest, *Rap1*-deficient liver samples also showed enrichment in gene sets involved in immune response pathways and in cell adhesion and cell-cell interactions (i.e., ECM receptor interaction and focal adhesion) (Table S6). By using qPCR in liver samples, we validated some of the differentially expressed genes involved in metabolism, including epidermal growth factor receptor (*Egfr*), insulin-like growth factor-binding protein 2 (*Igfbp2*), leptin receptor (*Lepr*), and insulin growth factor 1 (*Igf1*) (Figure S4A). We also found a 50% decrease in *Pgc1α* expression in the liver of *Rap1*-deficient mice, in agreement with previous results obtained with MEFs (Martinez et al., 2010).

Interestingly, when both the differentially expressed genes (transcriptome) and proteins (iTRAQ) in liver samples from female mice were analyzed by Ingenuity Systems Pathway analysis (Ingenuity IPA software), we found the same top-ten pathways affected in both sets of samples, namely fatty acid metabolism, xenobiotic metabolism, glycolysis and gluconeogenesis, bile acid biosynthesis, tryptophan, propanoate, linoleic, androgen/estrogen, pyruvate, and the metabolism of arachnoid acid (Figure S4B). Notably, both types of analyses indicated altered expression of a large number of PPARα target genes in the *Rap1*-deficient obese females (Figure S5), pinpointing to deregulation of PPARα as one of the key events associated with *Rap1* deficiency. In this regard, the PPAR signaling pathway is known to regulate a plethora of genes important for diverse cellular functions, including metabolism, cell proliferation, cell differentiation, apoptosis, and immune response.

### Decreased mRNA and Protein Expression of *Pparα* and *Pgc1α* in *Rap1*-Deficient Livers

**Figure 6:**
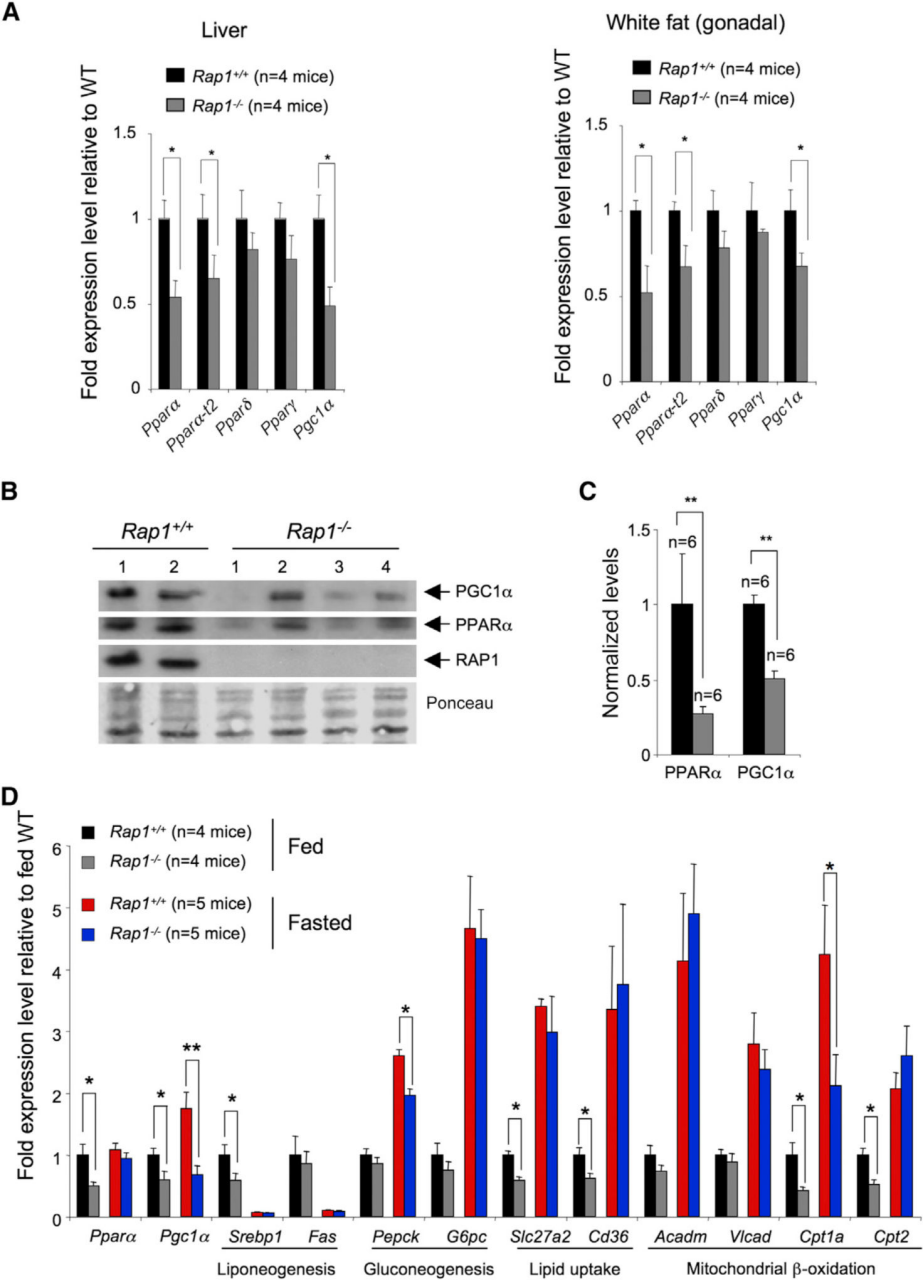
RAP1 Regulates Expression of *Pparα* and *Pgc1α* (A) qPCR analysis of the indicated genes in liver and gonadal white fat from 10-week-old wild-type and *Rap1* knockout females. Results are normalized to fed wild-type mean values. (B) Western blotting analysis of PGC1α, PPARα, and RAP1 protein levels in liver samples from wild-type and *Rap1* knockout females. (C) Quantification of PGC1α and PPARα protein levels in liver. Results are normalized to wild-type mean values. (D) qPCR analysis of the indicated genes in liver samples from 10-week-old wild-type and *Rap1* knockout females, which were either fed or after 24 hr fasting. The metabolic processes affected by the analyzed genes are indicated. Results are normalized to wild-type cells. Sample size (n) is indicated in each case. Error bars represent SD. Statistical significance was determined by two-tailed Student’s t test. *p < 0.05; **p < 0.01. See also Figures S3–S6.

Given the key role of the PPAR pathway in the regulation of metabolism together with the fact that this pathway was significantly deregulated in liver and white fat from young *Rap1*-deficient females before the onset of obesity, as well as in the obese mice, we next set out to study the expression of the three subtypes of PPARs, namely *Pparα, Pparγ*, and *Pparδ/β*, as well as their cofactor *Pgc1α*, in both liver and gonadal white fat samples from young 10-week-old female mice by using qPCR (Figure 6A). No significant differences in Pparγ and *Pparδ/β* expression were found between genotypes in both tissues at 10 weeks of age (Figure 6A). Interestingly, the levels of *Pparα* (NCBI RefSeq NM_011144) and *Pgc1α* were reduced by approximately 50% in liver and white fat from *Rap1*-deficient mice compared to the controls before the onset of obesity (Figure 6A). The levels of *Pparα* transcript variant 2 (*Pparα*-t2) (NCBI RefSeq NM_001113418) were also significantly reduced in both tissues from *Rap1*-deficient mice (Figure 6A). By using western blotting analysis, we confirmed a 0.5-fold reduction in PPARα and PGC1α protein levels in liver samples from *Rap1*-deficient mice (Figures 6B and 6C). These results suggest that deregulation of PPARα/PGC1α is one of the initial events that may trigger the transcriptional changes in both liver and white fat leading to obesity in *Rap1*-deficient mice.

### Defective Expression of PPARα Target Genes in *Rap1*-Deficient Livers upon Fasting

Given the observed deregulated expression of *Pparα* and its cofactor *Pgc1α* in *Rap1*-deficient tissues, we next set out to address the expression of several known PPARα downstream targets in young (10 weeks old; before the onset of obesity) females of both genotypes, which were either fed or fasted for 24 hr. Fasting is known to downregulate transcription of lipogenic genes in the liver and to upregulate genes involved in gluconeogenesis, lipid transport/uptake, and fatty acid oxidation, in this manner ensuring an adequate supply of substrates that can be metabolized by other tissues (Yoon et al., 2001). In particular, *Pgc1α* expression is induced by fasting and serves as a transcriptional booster to augment the capacity of metabolic adaptation to activate gluconeogenesis and fatty acid oxidation (Yoon et al., 2001). In contrast to the upregulation of *Pparα* gene expression observed in fasted SV129 wild-type (Kersten et al., 1999), we did not observe differences in the *Pparα* expression levels between fed and fasted states in our wild-type mice (Figure 6D).

Under nonfasting conditions, we confirmed decreased expression of *Pparα* and *Pgc1α* in liver samples from 10-week-old *Rap1*-deficient female mice (Figure 6D). We also observed decreased expression of a key regulator of lipogenesis, the sterol regulatory element-binding transcription factor 1 (*Srebp1*) (Figure 6D) (Horton et al., 2002; Liang et al., 2002). Interestingly, *Pparα*-deficient mice fed ad libitum also show decreased expression of *Srebp1c* (Hebbachi et al., 2008), in line with decreased *Pparα* expression in *Rap1*-deficient livers. Consistent with lower levels of PPARα/PGC1α in *Rap1*-deficient livers, we also observed decreased levels of the PPARα/PGC1α downstream targets SLC27a2 (solute carrier family 27) and CD36 (cluster of differentiation 36), which are important to transfer fatty acids across the cell membrane (Martin et al., 1997; Motojima et al., 1998; Rakhshandehroo et al., 2009). Finally, *Rap1*-deficient livers also showed decreased expression of *Cpt1* and *Cpt2* (carnitine palmitoyltransferase 1 and 2, respectively), which allow the transport of long-chain acyl-coenzyme A (CoA) across the inner mitochondrial membrane to enter the fatty acid β-oxidation pathway (Kersten et al., 1999; Rakhshandehroo et al., 2010) (Figure 6D). No differences were observed between genotypes in key regulators of gluconeogenesis (phosphoenol pyruvate carboxykinase [*Pepck*] and glucose-6-phosphatase [*G6Pase*]) (Figure 6D). Together, these results indicate that lipid accumulation in *Rap1*-deficient mice is likely to be the consequence of a reduced capacity for fatty acid import and utilization.

Upon fasting, we confirmed decreased liver transcription of genes involved in lipogenesis (*Srebp1*; fatty acid synthase [*Fas*]) and increased transcription of genes involved in gluconeogenesis (*Pepck, G6Pase*), lipid uptake (*Cd36, Slc27a2*), and mitochondrial β-oxidation (medium-chain and very long-chain acyl-CoA dehydrogenase, *Acadm* and *Vlcad*; as well as *Cpt1a* and *Cpt2*) (Figure 6D) in mice of both genotypes. The induction of PEPCK was lower in *Rap1*-deficient samples compared to wild-type (Figure 6D). Similarly, the induction of *Cpt1a* was lower in *Rap1*-deficient livers compared to wild-type controls (Figure 6D). Importantly, whereas the expression of *Pgc1α* was increased upon fasting in wild-type livers, *Rap1*-deficient livers failed to upregulate *Pgc1α* (Figure 6D), thus indicating a defective *Pgc1α* response in the liver as a consequence of fasting associated with *Rap1* deficiency.

In summary, gene expression profiling reveals that some PPARα target genes are affected by *Rap1* deletion in fed and fasted states (i.e., *Cpt1*), others only in the fed state (i.e., *Slc27a2, Cd36*, and *Cpt2*), and others only in the fasted state (i.e., *Pepck*). In contrast, the expression of other PPARα target genes, such as *Acadm, Vlcad, Fgf21, Cyp4a10*, and *Cyp4a14*, was not affected in *Rap1*-deficient mice in fed or fasted states (Figures 6D and S6A). These results may suggest that *Rap1* deletion does not fully abolish *Pparα* expression. On the other hand, they may suggest the convergence of different regulatory pathways toward the regulation of the expression of a single PPARα target gene.

In order to investigate whether RAP1 might regulate other metabolically important hepatic transcription factors, we performed gene expression profile analysis in liver samples of young females (10 weeks old) that had been fasting for 24 hr. Genes expressed in mouse liver were obtained from Barcode server with a consensus proportion of 0.95 (McCall et al., 2011). Transfac database annotations were employed to retrieve those genes described as transcription factors. The resulting gene set was tested by GSEA of *Rap1*-deficient versus wild-type mice. GSEA rendered a nonsignificant FDR value (0.49), demonstrating that this gene set was not deregulated in *Rap1*^−/−^ livers. However, out of the 66 transcription factors tested, *Pgc1α* was the most downregulated in *Rap1*^−/−^ liver compared to wild-type liver, being located in the first position of the wild-type side of GSEA ranking and showing the highest absolute values for the enrichment score and the log fold change (Figure S6B). These results underscore the specificity of RAP1 in the regulation of PPARα/PGC1α axis in the liver, which cannot be extended to every hepatic transcription factor.

### RAP1 Binds to *Pparα* and *Pgc1α* Loci

**Figure 7:**
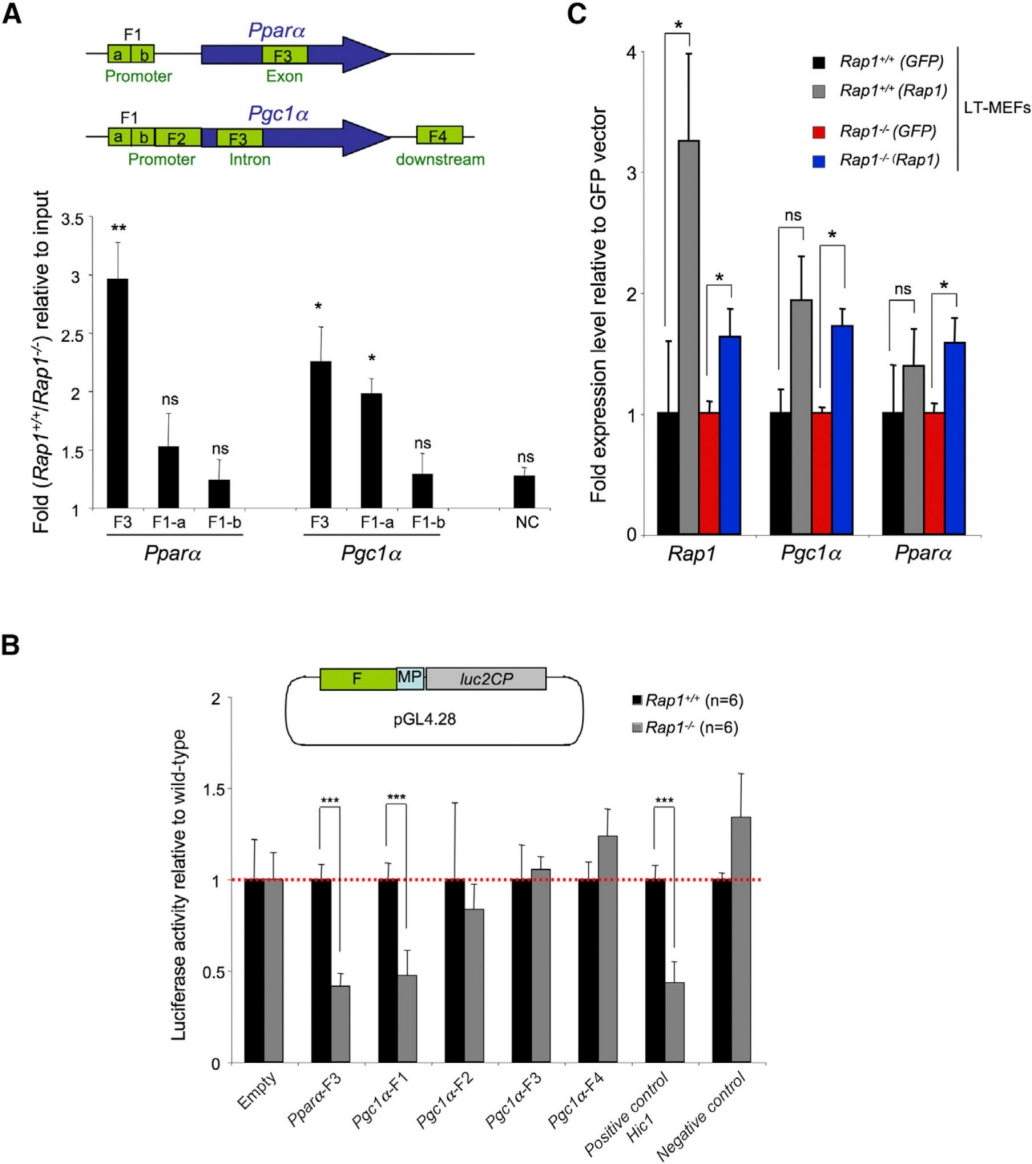
RAP1 Protects from Obesity through Regulating the Expression of *Pparα* and *Pgc1α* (A) ChIP of RAP1 and qPCR of different genomic DNA regions of *Pparα* and *Pgc1α* loci in wild-type and *Rap1*^−/−^ liver samples. A schematic representation of the analyzed fragments is depicted. F1 and F2 in *Pparα* and in *Pgc1α* loci were chosen based on the observed enrichment in regulatory elements in the upstream regulatory region of each gene (see Figure S7). F1-a and F1-b refer to different parts of F1. F3 and F4 in *Pparα* and in *Pgc1α* contain RAP1-binding peaks previously identified by ChIP-seq analysis (Martinez et al., 2010). Values correspond to the ratio between the percent immunoprecipitated DNA with respect to the input in the wild-type and *Rap1*^−/−^ liver samples. Three independent mice were analyzed per genotype. (B) Luciferase activity in wild-type and *Rap1* knockout LT-immortalized MEFs. A genomic fragment within *Pparα*-coding sequence (F3), two genomic fragments within the *Pgc1α* promoter (F1 and F2), one fragment located in a *Pgc1α* intron (F3), and other fragments downstream the *Pgc1α* gene (F4) were cloned upstream of a minimal promoter driving luciferase expression (schematic representation as in A). A genomic fragment within the *Hic1* locus and an aleatory-chosen genomic fragment not identified in ChIP-seq were used as positive and negative control, respectively (Martinez et al., 2010). The constructs were subsequently transfected into LT-immortalized MEFs. Results were normalized to the activity obtained in cells transfected with the empty vector. (C) *Rap1* transgenic expression in *Rap1*^−/−^ immortalized MEFs rescues *Pgc1α* and *Pparα* expression. LT-immortalized *Rap1*^+/+^ and *Rap1*^−/−^ MEFs were electrophorated with PTT3-RAP1 vector and with the vector harboring GFP as a negative control. Results are normalized with regards to expression levels in cells expressing GFP. Error bars represent SD. Statistical significance was determined by two-tailed Student’s t test. *p < 0.05; **p < 0.01; ***p < 0.001. See also Figure S7.

By using ChIP-seq in wild-type and *Rap1*-deficient MEFs, we previously demonstrated that RAP1 binds to intragenic sites within the *Pgc1α* and *Pparα* genes (Martinez et al., 2010) (this site is referred to as F3 in Figure 7A) (see Table S7 for genomic coordinates). To address whether RAP1 binds to these sites also in the liver, we performed ChIP analysis in *Rap1*-deficient and wild-type freshly isolated liver samples using an anti RAP1 antibody (Experimental Procedures). ChIP analysis followed by qPCR in fresh liver samples demonstrated that RAP1 binds to the intragenic F3 region in *Pparα* and *Pgc1α* genes in the liver. In addition to the peaks identified by ChIP-seq, we also tested RAP1 binding to *Pparα* and *Pgc1α* promoter regions. In particular, upon browsing *Pparα* and *Pgc1α* upstream regulatory regions around the transcription start site (TSS), we identified a region (F1, Figures 7A and S7) in both genes that was enriched in regulatory elements (Rosenbloom et al., 2013) and designed primers to amplify two fragments within F1 (F1-a and F1-b) (Experimental Procedures). qPCR on the immunoprecipitated DNA showed that RAP1 also binds to F1-a in *Pgc1α* promoter. RAP1 binding, however, was not detected at F1-b in *Pgc1α* promoter or the F1-a/F1-b in *Pparα* promoter (Figure 7A). These results indicate that RAP1 is recruited to *Pparα* and *Pgc1α* loci, supporting that Rap1 is involved in their transcriptional regulation.

### RAP1 Regulates *Pparα* and *Pgc1α* Transcription

To address whether RAP1 is involved in *Pparα* and *Pgc1α* transcriptional regulation, we cloned different DNA fragments belonging to the *Pparα* (F3 in Figure 7A; Figure S7) and *Pgc1α* loci (F1–F4 in Figure 7A; Figure S7) upstream of a minimal promoter driving luciferase expression (Experimental Procedures). F3 in *Pparα* locus corresponded to previously identified RAP1-binding peaks by ChIP-seq analysis (Martinez et al., 2010). F1 and F2 in *Pgc1α* locus corresponded to the *Pgc1α* promoter region, and F3 and F4 corresponded to previously identified RAP1-binding peaks by ChIP-seq analysis (Martinez et al., 2010), one located in intron 2 within *Pgc1α* ORF (F3) and the other located 6.5 kb downstream the *Pgc1α* gene (F4) (Figures 7A and 7B; Table S7). The different constructs were then transfected into *Rap1^+/+^* and *Rap1*^−/−^ LT-immortalized MEFs, and luciferase activity was measured after 48 hr (Figure 7B). Interestingly, luciferase activity was significantly decreased in *Rap1*^−/−^ cells compared to wild-type cells transfected with *Pparα*-F3 and *Pgc1α*-F1, which were found to contain a RAP1-binding site by ChIP assay (Figure 7A). No differences were observed in F2–F4, with the empty vector or with the vector harboring a genomic fragment used as negative control (Figure 7B). A fragment within the *Hic1* locus previously shown to contain RAP1-dependent enhancer activity was used as positive control (Martinez et al., 2010). These results suggest that *Pparα*-F3 and *Pgc1α*-F1 have RAP1-dependent enhancer activity and strongly suggest a role for RAP1 in *Pparα* and *Pgc1α* transcriptional regulation.

We previously showed that *Rap1*-deficient MEFs have a decreased *Pgc1α* expression (Martinez et al., 2010). To demonstrate that decreased PGC1α and PPARα levels are due to *Rap1* deficiency, a *Rap1*-containing vector was transfected into immortalized wild-type and *Rap1*-deficient MEFs. A GFP-containing vector was also transfected as negative control as well as to estimate transfection efficiency. The expression levels of *Ppparα* and *Pgc1α* were then determined 48 hr after transfection. We found a significant recovery in *Ppparα* and *Pgc1α* transcription levels in *Rap1*-deficient MEFs upon RAP1 transgenic expression, indicating a direct role of RAP1 in the transcriptional regulation of these genes (Figure 7C).

## DISCUSSION

By generating a whole-body *Rap1*-deficient mouse model, we show here that the mammalian telomere-binding protein RAP1 is dispensable for mouse development and adult viability, in contrast to that previously reported by Teo et al. (2010) and in agreement with Sfeir et al. (2010). In agreement with our previous findings that RAP1 binds throughout chromosome arms (Martinez et al., 2010), we find a role for RAP1 in the transcriptional regulation of pathways involved in postnatal cellular energy metabolism. In line with this, adult *Rap1*-deficient mice are obese and show abnormal accumulation of fat in abdominal tissues, concomitant with hepatic steatosis and glucose resistance.

In particular, by using both gene expression and iTRAQ analysis of *Rap1*-deficient liver and white fat tissues, we find a significant deregulation of the PPAR signaling pathway, a key player in the regulation of energy homeostasis. By using ChIP analysis, we further demonstrate that RAP1 binds to *Pparα* and *Pgc1α* loci in liver, and that can regulate transcription of *Pparα* and *Pgc1α*. In line with this, *Rap1*-deficient mice show decreased *Pparα* and *Pgc1α* expression and the subsequent deregulation of some of their target genes, leading to severe metabolic alterations that are in accordance with the early onset of obesity found in these mice.

We show here that *Rap1*-deficient mice also develop hepatic steatosis. In this regard, the PPARα/PGC1α complex is a key regulator of fatty acid oxidation (Kersten et al., 1999; Leone et al., 1999, 2005). CPT1, a target of PPARα/PGC1α, constitutes the rate-limiting step in fatty acid oxidation (Djouadi et al., 1998). Our results show that *Cpt1a* and *Cpt2* expression is decreased in *Rap1*-deficient livers compared to wild-type controls, suggesting that *Rap1*-deficient mice are defective in fatty acid catabolism. Oxidation of fatty acids in the liver is also tightly coupled to glucose synthesis (Yoon et al., 2001). In fasted animals, we find that the expression of the key gluconeogenic enzyme, PEPCK, is decreased in *Rap1*-deficient mice. Similarly, the expression of several fatty acid transporters, SLC27a2 and CD36, is also significantly decreased in *Rap1*-deficient livers. In summary, these findings place RAP1 as a key factor in the physiologic regulation of lipid homeostasis, through the PPARα/PGC1α regulatory pathway.

Further supporting a role for RAP1 in modulating PPARα/PGC1α, the phenotypes of *Rap1*-deficient mice are strikingly similar to those of *Pparα*-deficient mice. In particular, similar to *Rap1* deficiency, PPARα deficiency leads to a late onset of spontaneous obesity with a remarkable sexual dimorphism. As in the case of *Rap1* deficiency, PPARα abrogation leads to a more pronounced obesity phenotype in females than in males, although males also develop hepatic steatosis (Costet et al., 1998; Lee et al., 1995). Moreover, female mice deficient in PGC1α, the PPARα cofactor, also show increased body weight (Leone et al., 2005). Interestingly, it has been shown that PPARα has broad female-dependent repressive actions on hepatic genes involved in steroid metabolism and immunity. In particular, specific gene sets involved in steroid metabolism, as well as androgen and estrogen metabolism, have been shown to exhibit PPARα-dependent sexual dimorphism (Leuenberger et al., 2009). These genes are upregulated in *PPARα*-deficient females but remained unchanged in PPARα-*null* males compared to wild-type controls. We find here that the same pathways are upregulated in the liver of *Rap1*-deficient females compared to wild-type females (see Table S2), strongly supporting a role of RAP1 in sex-specific PPARα functions.

Telomere shortening in the context of telomerase-deficient mice was previously shown to repress *Pgc1α*/β and its downstream transcriptional network, leading to mitochondrial dysfunction (i.e., compromised OXPHOS and respiration, decreased ATP generation capacity, and increased oxidative stress). In particular, short/dysfunctional telomeres lead to increased p53 levels, which can bind to *Pgc1α* and *Pgc1β* promoters and repress their transcriptional expression (Sahin et al., 2011). In the setting of the severe metabolic changes induced by RAP1 deficiency, however, we did not observe the presence of short/dysfunctional telomeres in liver or brown fat, indicating that metabolic changes associated with RAP1 deficiency are independent of telomere dysfunction. This is further supported by clearly distinct mouse phenotypes associated with either telomerase deficiency or RAP1 deficiency. Telomerase-deficient mice show a dramatic reduction in lifespan, lower body weight, and decreased fat mass (Herrera et al., 1999; Lee et al., 1998; Sahin et al., 2011). In contrast, *Rap1* knockout mice show an obese phenotype and no differences in survival curves as compared to wild-type controls.

In conclusion, we demonstrate here that RAP1 serves as a transcriptional regulator that controls the capacity of downstream metabolic pathways critical for metabolic maturation. In its absence, female mice develop obesity, glucose intolerance, and hepatic steatosis. We propose that the *Rap1* null mutant mouse should serve as a useful murine model for studying the role of altered energy metabolism in obesity, diabetes, and hepatosteatosis.

## EXPERIMENTAL PROCEDURES

### Generation of Whole-Body *Rap1* Knockout Mice

*Rap1* knockout mice, *Rap1*^−/−^, were generated by crossing *Rap1^flox/flox^* mice (Martinez et al., 2010) with a transgenic mouse line carrying the *cre* recombinase under the control of the adenovirus *EIIa* promoter, *EIIA-cre* mice (Lakso et al., 1996). Intercrosses to heterozygous *Rap1*^+/−^ mice not harboring the *EIIA-cre* allele resulted in the removal of the *EIIA-cre* allele in the mouse colony arising from amplification. The genetic background of the mice was C57BL6/129SV (90%/10%). All mice were generated and maintained at the Animal Facility of the Spanish National Cancer Research Centre (CNIO) under specific pathogen-free conditions in accordance with the recommendation of the Federation of European Laboratory Animal Science Associations.

Mice were fed either a standard chow diet (Harlan Teklad 2018; 18% calories from fat) or, when indicated, a HFD (Research Diets D12451; 45% of total calories from fat) starting at 4 weeks of age. Trained personnel performed weekly observations of all mice. Upon detection of signs of morbidity, mice were closely inspected daily until application of Humane End Point criteria (http://dels.nas.edu/global/ilar/Guide).

### Serum Analysis

Glucose in serum was measured using Glucocard strips (A. Meranini Diagnostics). Insulin levels were determined by ELISA (Ultra Sensitive Mouse Insulin ELISA kit; Crystal Chem). Insulin sensitivity was evaluated by the HOMA-IR (fasting insulin [µU/ml] × fasting glucose [mg/dl]/405) and the QUICKI (1/(log(fasting insulin [µU/ml]) + log(fasting glucose [mg/dl]). Serum ALT, cholesterol, triglycerides, bilirubin, urea, creatinine, albumin, total proteins, lactate, phosphorous, and glucose were determined using ABX Pentra (Horiba Medical). Plasma-free fatty acid and ketone body levels were analyzed by in vitro enzymatic colorimetric method assays (NEFA-HR and Autokit 3-HB kits, respectively; Wako Chemicals). To perform the GTT and ITT, mice were i.p. injected, respectively, with 2 g of glucose/kg of body weight and 0.75 IU insulin/kg of body weight (Eli Lilly; Humalog Insulin). Tail blood glucose levels were measured with a glucometer at the required times after injection. Prior to the GTT, mice were subjected to 8 hr of fasting. Triglyceride content in liver samples was determined by colorimetric assay kit (Cayman Chemical).

### Gene Expression Analysis

Total RNA samples from liver and white fat tissues were analyzed on Agilent’s Mouse Genome DNA microarray following the manufacturer’s instructions. Images were quantified using Agilent Feature Extraction Software (v.10.1.1).

### Luciferase and ChIP Assays

Luciferase assay and ChIP assays were performed as previously described (Martinez et al., 2010).

## Supplementary Material

Document S1. Tables S1-S3 and S5

Table S6

Table S7

Figure S1

Figure S2

Figure S3

Figure S4

Figure S5

Figure S6

Figure S7

Table S4
