# Supplementary material for: RAP1 Protects from Obesity through Its Extratelomeric Role Regulating Gene Expression
Source: Cell Rep. Author manuscript; Available in PMC 2018 Apr 7. (PMC5889507; doi:10.1016/j.celrep.2013.05.030)
Supplement: Document S1. Tables S1-S3 and S5 [file NIHMS948947-supplement-36.pdf]

Table S1. Plasma Analysis of Metabolic Parameters in Male and Female Mice Subjected Either to Standard Diet or to a High-Fat Diet, Related to Figure 3
*, p=0.05; **, p<0.01.

	**Standard Diet**		**High fat diet**	
**MALES**	***Rap1***$^{+/+}$	***Rap1***$^{-/-}$	***Rap1***$^{+/+}$	***Rap1***$^{-/-}$
Weight (g)	42.1 ± 3.7	47.6 ± 2.5	44.9 ± 3.0	48.5 ± 1.5
Albumin (g/L)	35.9 ± 1.1	36.1 ± 2.7	35.1 ± 1.0	34.7 ± 0.6
Bilirubin (μmol/L)	2.6 ± 0.4	3.6 ± 1.2	5.9 ± 3.0	2.5 ± 0.2
Calcium (mmol/L)	2.3 ± 0.1	2.1 ± 0	2.2 ± 0.1	2.2 ± 0
Phosphorous (mmol/L)	3.2 ± 0.3	4.0 ± 0.5	2.5 ± 0.1	2.3 ± 0.1
Total proteins (g/dL)	7.2 ± 0.2	6.9 ± 0.2	6.4 ± 0.3	6.3 ± 0.2
Urea (mg/dL)	64.8 ± 9.0	64.0 ± 5.2	56.6 ± 3.1	54.9 ± 4.1
Creatinine (μmol/L)	13.1 ± 4.1	11.3 ± 1.3	10.3 ± 1.6	12.1 ± 1.3
Cholesterol (mg/L)	146.9 ± 6.7	132.4 ± 25.4	194.4 ± 25.9	181.3 ± 8.3
HDL (mg/dL)	53.0 ± 8.0	65.2 ± 2.6	72.3 ± 4.4	68.5 ± 2.9
LDL (mg/dL)	10.0 ± 0.7	12.5 ± 1.3	24.8 ± 4.3	21.8 ± 2.6
Triglycerides (mg/dL)	123.0 ± 13.1	121 ± 9.0	124.3 ± 11.3	114.4 ± 8.4
ALT (U/L)	35.88 ± 3.0	49.64 ± 9.2	195.12 ± 87	111.6 ± 28
Glucose (mmol/L)	5.4 ± 1.5	5.1 ± 0.2	5.4 ± 1.5	4.0 ± 0.8

	**Standard Diet**		**High fat diet**	
**FEMALES**	***Rap1***$^{+/+}$	***Rap1***$^{-/-}$	***Rap1***$^{+/+}$	***Rap1***$^{-/-}$
Weight (g)	28.9 ± 3.1	49.5 ± 4.6 **	32.6 ± 3.5	44.0 ± 2.7**
Albumin (g/L)	32.6 ± 2.2	34.2 ± 2.5	34.0 ± 1.9	35.7 ± 1.0
Bilirubin (μmol/L)	4.0 ± 0.9	1.7 ± 0.2	2.6 ± 0.2	2.6 ± 0.2
Calcium (mmol/L)	2.1 ± 0.1	2.3 ± 0	2.3 ± 0.1	2.4 ± 0.1
Phosphorous (mmol/L)	3.2 ± 0.3	2.7 ± 0.2	2.1 ± 0.2	2.5 ± 0.3
Total proteins (g/dL)	6.1 ± 0.6	6.5 ± 0.1	6.5 ± 0.3	6.7 ± 0.2
Urea (mg/dL)	60.9 ± 3.6	57.5 ± 1.8	57.8 ± 2.9	58.8 ± 2.6
Creatinine (μmol/L)	15.8 ± 3.0	7.9 ± 1.2	13.6 ± 1.8	12.1 ± 2.1
Cholesterol (mg/L)	85.6 ± 18.6	118.6 ± 4*	112.4 ± 12	159 ± 10**
HDL (mg/dL)	41.3 ± 8.9	44.3 ± 4.8	51.0 ± 5.1	62.5 ± 1.4
LDL (mg/dL)	9.6 ± 0.8	11.6 ± 1.6	13.3 ± 0.6	18.6 ± 1.3**

Triglycerides (mg/dL)	136.7 ± 10	120.7 ± 7.5	115.5 ± 5.8	111.7 ± 5.6
ALT (U/L)	54.1 ± 14.6	129.53 ± 38**	58.55 ± 20	187 ± 38**
Glucose (mmol/L)	4.1 ± 1.4	5.5 ± 0.2	5.0 ± 0.5	4.3 ± 0.6

Table S2. Significantly Enriched Gene Sets Found in the Livers of 10-Week-Old *Rap1*$^{-/-}$ Females, Related to Figure 6

FDR: False Discovery Rate, NES: Normalized Enrichment Score. FDR<0.25.

Gene Set Name	# Genes	NES	FDR q-val	Source	Rab1 -/- status
**BIOSYNTHESIS OF STEROIDS**	23	2.5169961	0	KEGG	UP
**DRUG METABOLISM - CYTOCHROME P450**	75	2.351679	0	KEGG	UP
**ANDROGEN AND ESTROGEN METABOLISM**	34	2.1567147	0	KEGG	UP
**CHOLESTEROL BIOSYNTHESIS**	19	2.4279442	0	REACTOME	UP
**METABOLISM OF XENOBIOTICS BY CYTOCHROME P450**	66	2.1008756	8.69E-04	KEGG	UP
**BILE ACID BIOSYNTHESIS**	29	2.0114846	9.04E-04	KEGG	UP
**TRYPTOPHAN METABOLISM**	42	2.0153143	0.001017382	KEGG	UP
**PROPANOATE METABOLISM**	29	2.0290937	0.001162723	KEGG	UP
**DRUG METABOLISM - OTHER ENZYMES**	46	2.0367715	0.00135651	KEGG	UP
**VALINE, LEUCINE AND ISOLEUCINE DEGRADATION**	45	2.0415428	0.001379811	KEGG	UP
**BUTANOATE METABOLISM**	40	1.9751942	0.001500798	KEGG	UP
**RETINOL METABOLISM**	62	1.8931497	0.004285994	KEGG	UP
**BIOLOGICAL OXIDATIONS**	119	2.0317812	0.007228908	REACTOME	UP
**ACTIVATION OF THE PRE-REPLICATIVE COMPLEX**	29	1.9477711	0.017360628	REACTOME	UP
**PYRIMIDINE METABOLISM**	89	1.7511019	0.01925754	KEGG	UP
**HISTIDINE METABOLISM**	27	1.7580942	0.019640766	KEGG	UP
**SPHINGOLIPID METABOLISM**	36	1.7398285	0.020183748	KEGG	UP
**ACTIVATION OF ATR IN RESPONSE TO REPLICATION STRESS**	35	1.9065479	0.020955402	REACTOME	UP
**PORPHYRIN AND CHLOROPHYLL METABOLISM**	32	1.7142601	0.024497299	KEGG	UP
**OXIDATIVE PHOSPHORYLATION**	110	1.699594	0.025012853	KEGG	UP
**FATTY ACID METABOLISM**	43	1.703376	0.025527395	KEGG	UP
**PYRUVATE METABOLISM**	40	1.681831	0.02740653	KEGG	UP
**P450 HYDROXYLATIONS**	17	1.8235364	0.029125301	REACTOME	UP
**P450 DEHYDROGENATION OF ALKANES TO FORM ALKENES**	19	1.8323857	0.030639747	REACTOME	UP
**DNA REPLICATION**	96	1.8384043	0.03243205	REACTOME	UP
**DNA REPLICATION PRE-INITIATION**	75	1.8597062	0.03294014	REACTOME	UP
**PURINE METABOLISM**	144	1.6597061	0.0333265	KEGG	UP
**FATTY ACIDS**	15	1.7806872	0.04325612	REACTOME	UP
**M/G1 TRANSITION**	61	1.7169871	0.04422614	REACTOME	UP
**PHASE 1 - FUNCTIONALIZATION OF COMPOUNDS**	71	1.7568594	0.04476819	REACTOME	UP
**CELL CYCLE CHECKPOINTS**	116	1.7089447	0.045446668	REACTOME	UP
**ELECTRON TRANSPORT CHAIN**	62	1.7455995	0.046201337	REACTOME	UP
**LIPID AND LIPOPROTEIN METABOLISM**	180	1.7636501	0.046276826	REACTOME	UP
**BRANCHED-CHAIN AMINO ACID CATABOLISM**	22	1.7193013	0.046503764	REACTOME	UP
**ASSEMBLY OF THE PRE-REPLICATIVE COMPLEX**	61	1.7319005	0.04792924	REACTOME	UP
**ORC1 REMOVAL FROM CHROMATIN**	68	1.6947447	0.048778545	REACTOME	UP
**G2/M CHECKPOINTS**	45	1.7210207	0.04892913	REACTOME	UP
**GLUTATHIONE CONJUGATION**	18	1.685878	0.049869407	REACTOME	UP
**ATP FORMATION**	21	1.6560509	0.050344132	REACTOME	UP
**DNA STRAND ELONGATION**	29	1.668273	0.05125659	REACTOME	UP

**FURTHER PLATELET RELEASATE**	37	1.6615132	0.05128681	REACTOME	UP
**FORMATION OF PLATELET PLUG**	116	1.6639029	0.051657442	REACTOME	UP
**EXOCYTOSIS OF ALPHA GRANULE**	70	1.6563364	0.052242845	REACTOME	UP
**TYROSINE METABOLISM**	36	1.601104	0.053438894	KEGG	UP
**CYTOCHROME P450**	25	1.6686112	0.0535614	REACTOME	UP
**HEMOSTASIS**	233	1.6699935	0.055531364	REACTOME	UP
**DNA REPLICATION**	35	1.5892345	0.05606858	KEGG	UP
**GLUTATHIONE METABOLISM**	53	1.584594	0.05608329	KEGG	UP
**COMPLEMENT AND COAGULATION CASCADES**	70	1.5644605	0.06346488	KEGG	UP
**UREA CYCLE AND METABOLISM OF AMINO GROUPS**	27	1.5336365	0.070784725	KEGG	UP
**ECM-RECEPTOR INTERACTION**	79	1.5421149	0.072123595	KEGG	UP
**GLYCINE, SERINE AND THREONINE METABOLISM**	44	1.5353991	0.07251592	KEGG	UP
**FORMATION OF FIBRIN CLOT (CLOTTING CASCADE)**	29	1.6077151	0.07336275	REACTOME	UP
**CYTOCHROME P450 - ARRANGED BY SUBSTRATE TYPE**	50	1.6014193	0.074300565	REACTOME	UP
**LINOLEIC ACID METABOLISM**	40	1.5079641	0.084434725	KEGG	UP
**G1/S TRANSITION**	109	1.5702939	0.0924462	REACTOME	UP
**NUCLEOTIDE METABOLISM**	82	1.564307	0.09304774	REACTOME	UP
**ELEVATION OF CYTOSOLIC CA++ LEVELS**	82	1.5488926	0.09932146	REACTOME	UP
**GLUCONEOGENESIS**	33	1.5512133	0.100048825	REACTOME	UP
**CDT1 ASSOCIATION WITH THE CDC6:ORC:ORIGIN COMPLEX**	55	1.5378649	0.10466529	REACTOME	UP
**NUCLEOTIDE EXCISION REPAIR**	40	1.4483975	0.12484158	KEGG	UP
**CITRATE CYCLE (TCA CYCLE)**	30	1.4495149	0.12825158	KEGG	UP
**FGFR4 LIGAND BINDING AND ACTIVATION**	15	-1.7794468	0.1311975	REACTOME	DOWN
**PENTOSE AND GLUCURONATE INTERCONVERSIONS**	18	1.4361193	0.1322497	KEGG	UP
**METABOLISM OF NITRIC OXIDE**	25	1.4967877	0.1385311	REACTOME	UP
**METHIONINE METABOLISM**	21	1.4033546	0.16125782	KEGG	UP
**INSULIN SYNTHESIS AND SECRETION**	54	1.4575654	0.16826625	REACTOME	UP
**CELL CYCLE, MITOTIC**	317	1.4583124	0.17213766	REACTOME	UP
**ORNITHINE METABOLISM**	53	1.4614093	0.1739477	REACTOME	UP
**NRAGE SIGNALS DEATH THROUGH JNK**	15	-1.6779176	0.17573065	REACTOME	DOWN
**AMINOACYL-TRNA BIOSYNTHESIS**	42	1.383633	0.17774573	KEGG	UP
**SELENOAMINO ACID METABOLISM**	22	1.3783097	0.1780914	KEGG	UP
**APC/C:CDH1 MEDIATED DEGRADATION OF CDC20 AND OTHER APC/C:CDH1 TARGETED PROTEINS IN LATE MITOSIS/EARLY G1**	71	1.4450498	0.17934485	REACTOME	UP
**COLLAGEN-MEDIATED ACTIVATION CASCADE**	16	1.4328508	0.18744712	REACTOME	UP
**CYCLIN A:CDK2-ASSOCIATED EVENTS AT S PHASE ENTRY**	64	1.4344866	0.18998687	REACTOME	UP
**DNA REPAIR**	98	1.4264888	0.19095255	REACTOME	UP
**PARKINSON'S DISEASE**	110	1.3624933	0.1911234	KEGG	UP
**AUTODEGRADATION OF CDH1 BY CDH1:APC/C**	63	1.4145646	0.19322754	REACTOME	UP
**CHYLOMICRON-MEDIATED LIPID TRANSPORT**	16	1.4162815	0.19504194	REACTOME	UP
**APC/C:CDC20 MEDIATED DEGRADATION OF SECURIN**	64	1.4187945	0.19619536	REACTOME	UP
**INTEGRIN CELL SURFACE INTERACTIONS**	91	1.4010003	0.20655149	REACTOME	UP

**CDC20:PHOSPHO-APC/C MEDIATED DEGRADATION OF CYCLIN A**	68	1.3953516	0.20984785	REACTOME	UP
**DOUBLE-STRAND BREAK REPAIR**	31	1.3887703	0.21332806	REACTOME	UP
**G-PROTEIN ACTIVATION**	25	1.3749077	0.22127932	REACTOME	UP
**G2/M TRANSITION**	97	1.3807743	0.22167584	REACTOME	UP
**MEMBRANE TRAFFICKING**	58	1.3684113	0.2217279	REACTOME	UP
**DUAL INCISION REACTION IN GG-NER**	21	1.3698521	0.2241191	REACTOME	UP
**FORMATION OF INCISION COMPLEX IN GG-NER**	21	1.375531	0.22492968	REACTOME	UP
**PPAR SIGNALING PATHWAY**	74	1.3206327	0.23484382	KEGG	UP
**ALANINE AND ASPARTATE METABOLISM**	29	1.3252659	0.23598894	KEGG	UP
**METABOLISM OF VITAMINS AND COFACTORS**	46	1.3481523	0.24488553	REACTOME	UP
**MISMATCH REPAIR**	22	1.3085029	0.24567536	KEGG	UP

Table S3. Significantly Enriched Gene Sets Found in Gonadal White Fat Tissue of *Rap1*$^{-/-}$ Females, Related to Figure 6

FDR: False Discovery Rate, NES: Normalized Enrichment Score. FDR<0.25.

Gene Set Name	# Genes	NES	FDR q-val	Source	Rab1 -/- status
**ECM-RECEPTOR INTERACTION**	79	2.004533	0.00552448	KEGG	UP
**PYRUVATE METABOLISM**	40	-1.9658341	0.009929905	KEGG	DOWN
**BLADDER CANCER**	42	1.9007902	0.013026089	KEGG	UP
**MAPK SIGNALING PATHWAY**	257	1.8657416	0.016694758	KEGG	UP
**FOCAL ADHESION**	190	1.8328364	0.020281324	KEGG	UP
**PROPANOATE METABOLISM**	29	-1.7186533	0.05040007	KEGG	DOWN
**PPAR SIGNALING PATHWAY**	74	-1.727355	0.05786784	KEGG	DOWN
**CITRATE CYCLE (TCA CYCLE)**	30	-1.6800555	0.059602737	KEGG	DOWN
**ELECTRON TRANSPORT CHAIN**	62	-1.8369874	0.06062995	REACTOME	DOWN
**GLUCOSE METABOLISM**	77	-1.7885938	0.061885286	REACTOME	DOWN
**BIOSYNTHESIS OF UNSATURATED FATTY ACIDS**	30	-1.6574196	0.06351131	KEGG	DOWN
**GLYCOLYSIS**	22	-1.8915594	0.06478297	REACTOME	DOWN
**TGF-BETA SIGNALING PATHWAY**	85	1.7114409	0.07026618	KEGG	UP
**GRAFT-VERSUS-HOST DISEASE**	21	-1.6271291	0.07157563	KEGG	DOWN
**BASAL CELL CARCINOMA**	55	1.6884203	0.07370097	KEGG	UP
**OXIDATIVE PHOSPHORYLATION**	110	-1.7316537	0.076298594	KEGG	DOWN
**HEDGEHOG SIGNALING PATHWAY**	53	1.6672674	0.080229335	KEGG	UP
**METABOLISM OF CARBOHYDRATES**	100	-1.7306147	0.08141958	REACTOME	DOWN
**METABOLISM OF BILE ACIDS AND BILE SALTS**	47	-1.6955582	0.08866284	REACTOME	DOWN
**COMPLEMENT AND COAGULATION CASCADES**	70	-1.5749704	0.09517886	KEGG	DOWN
**GLYCOLYSIS / GLUCONEOGENESIS**	53	-1.7444655	0.09906602	KEGG	DOWN
**G-PROTEIN ACTIVATION**	25	1.8571392	0.10200319	REACTOME	UP
**COLLAGEN ADHESION VIA ALPHA 2 BETA 1 GLYCOPROTEIN**	16	1.7720693	0.13963076	REACTOME	UP
**P53 SIGNALING PATHWAY**	65	1.562774	0.14037709	KEGG	UP
**FGFR1 LIGAND BINDING AND ACTIVATION**	20	1.6028283	0.1410056	REACTOME	UP
**ALPHA-LINOLENIC ACID METABOLISM**	16	1.5832515	0.14237046	KEGG	UP
**FURTHER PLATELET RELEASATE**	37	1.6184621	0.1443063	REACTOME	UP
**PROTEASOME**	41	-1.5062642	0.14546703	KEGG	DOWN
**BASIGIN INTERACTIONS**	15	1.5896456	0.1458923	REACTOME	UP
**FGFR2 LIGAND BINDING AND ACTIVATION**	19	1.6037223	0.15209194	REACTOME	UP
**WNT SIGNALING PATHWAY**	146	1.5635965	0.15346472	KEGG	UP
**INTEGRIN CELL SURFACE INTERACTIONS**	91	1.6207877	0.15701488	REACTOME	UP
**COLORECTAL CANCER**	85	1.5840312	0.15961584	KEGG	UP
**ENDOGENOUS STEROLS**	18	-1.6016139	0.16369729	REACTOME	DOWN
**EXOCYTOSIS OF ALPHA GRANULE**	70	1.5255255	0.17436944	REACTOME	UP
**COLLAGEN-MEDIATED ACTIVATION CASCADE**	16	1.6214712	0.1757661	REACTOME	UP

**CELL SURFACE INTERACTIONS AT THE VASCULAR WALL**	93	1.5353462	0.17903456	REACTOME	UP
**OPIOID SIGNALLING**	82	1.5282247	0.17944291	REACTOME	UP
**CYCLIN D ASSOCIATED EVENTS IN G1**	17	1.5355306	0.18994336	REACTOME	UP
**FGFR LIGAND BINDING AND ACTIVATION**	23	1.6278551	0.19034383	REACTOME	UP
**FORMATION OF PLATELET PLUG**	116	1.6787493	0.19109751	REACTOME	UP
**FGFR2C LIGAND BINDING AND ACTIVATION**	15	1.5422461	0.19196635	REACTOME	UP
**PARKINSON'S DISEASE**	110	-1.451378	0.19311035	KEGG	DOWN
**ARACHIDONIC ACID METABOLISM**	69	1.484856	0.19386934	KEGG	UP
**CELL ADHESION MOLECULES (CAMS)**	110	1.4999945	0.1983287	KEGG	UP
**G1 PHASE**	17	1.5028735	0.19880383	REACTOME	UP
**AMINE-DERIVED HORMONES**	16	1.5452377	0.200567	REACTOME	UP
**PEPTIDE LIGAND-BINDING RECEPTORS**	65	1.6365684	0.20280737	REACTOME	UP
**B CELL RECEPTOR SIGNALING PATHWAY**	65	1.4876983	0.20352997	KEGG	UP
**ELEVATION OF CYTOSOLIC CA++ LEVELS**	82	1.4868823	0.2037268	REACTOME	UP
**N-GLYCAN DEGRADATION**	15	1.5073115	0.20423242	KEGG	UP
**NRAGE SIGNALS DEATH THROUGH JNK**	15	1.4927717	0.20439309	REACTOME	UP
**INTEGRATION OF ENERGY METABOLISM**	54	-1.4255652	0.20896266	REACTOME	DOWN
**METABOLISM OF NITRIC OXIDE**	25	-1.4349115	0.21015264	REACTOME	DOWN
**IMMUNOREGULATORY INTERACTIONS BETWEEN A LYMPHOID AND A NON-LYMPHOID CELL**	46	1.4764822	0.21061532	REACTOME	UP
**MELANOGENESIS**	100	1.4620317	0.21549417	KEGG	UP
**PHASE 1 - FUNCTIONALIZATION OF COMPOUNDS**	71	-1.4004295	0.21579671	REACTOME	DOWN
**HEMOSTASIS**	233	1.69418	0.21611644	REACTOME	UP
**METABOLISM OF WATER-SOLUBLE VITAMINS AND COFACTORS**	46	-1.4395648	0.21927734	REACTOME	DOWN
**LIPID AND LIPOPROTEIN METABOLISM**	180	-1.5230713	0.22054918	REACTOME	DOWN
**CITRIC ACID CYCLE (TCA CYCLE)**	18	-1.5427071	0.22126439	REACTOME	DOWN
**BRANCHED-CHAIN AMINO ACID CATABOLISM**	22	-1.4054058	0.22159475	REACTOME	DOWN
**CYTOCHROME P450 - ARRANGED BY SUBSTRATE TYPE**	50	-1.4619826	0.22325319	REACTOME	DOWN
**METABOLISM OF VITAMINS AND COFACTORS**	46	-1.4479984	0.22469375	REACTOME	DOWN
**DEGRADATION OF BETA-CATENIN BY THE DESTRUCTION COMPLEX**	55	-1.4727879	0.22477409	REACTOME	DOWN
**CHYLOMICRON-MEDIATED LIPID TRANSPORT**	16	-1.484501	0.22968222	REACTOME	DOWN
**CLASS A/1 (RHODOPSIN-LIKE RECEPTORS)**	65	1.6407377	0.23283313	REACTOME	UP
**MEMBRANE TRAFFICKING**	58	1.4549385	0.23500216	REACTOME	UP
**CYTOKINE-CYTOKINE RECEPTOR INTERACTION**	237	1.4410808	0.23852608	KEGG	UP
**P450 DEHYDROGENATION OF ALKANES TO FORM ALKENES**	19	-1.3733454	0.24052711	REACTOME	DOWN
**FORMATION OF FIBRIN CLOT (CLOTTING CASCADE)**	29	-1.4933748	0.24174394	REACTOME	DOWN

Table S5. Overrepresentation Analysis of Gene Ontology Terms for the Significantly Deregulated Genes in *Rap1*$^{-/-}$ Liver, Related to Figure 6

FDR: False Discovery Rate. FDR<0.05.

GO BIOLOGICAL PROCESS			
**GO term ID**	**GO term description**	**FDR**	**Rap1 -/- status**
GO:0006082	organic acid metabolic process	3.96E-03	UP
GO:0019752	carboxylic acid metabolic process	3.96E-03	UP
GO:0044255	cellular lipid metabolic process	3.96E-03	UP
GO:0008202	steroid metabolic process	1.17E-02	UP
GO:0006631	fatty acid metabolic process	1.23E-02	UP
GO:0006629	lipid metabolic process	1.62E-02	UP
GO:0008203	cholesterol metabolic process	4.30E-02	UP
GO:0007623	circadian rhythm	4.45E-02	DOWN
GO:0010033	response to organic substance	4.45E-02	DOWN

GO CELLULAR COMPONENT			
**GO term ID**	**GO term description**	**FDR**	**Rap1 -/- status**
GO:0005783	endoplasmic reticulum	4.73E-05	UP
GO:0019866	organelle inner membrane	1.12E-02	UP
GO:0005792	microsome	2.13E-02	UP
GO:0042175	nuclear outer membrane-endoplasmic reticulum membrane network	2.13E-02	UP
GO:0042598	vesicular fraction	2.20E-02	UP
GO:0000267	cell fraction	2.27E-02	UP
GO:0009986	cell surface	4.29E-02	UP
GO:0044429	mitochondrial part	4.29E-02	UP
GO:0031225	anchored to membrane	4.29E-02	UP
GO:0046658	anchored to plasma membrane	4.29E-02	UP
GO:0005579	membrane attack complex	0.0485849	DOWN
GO:0005783	endoplasmic reticulum	0.0485849	DOWN

GO MOLECULAR FUNCTION			
**GO term ID**	**GO term description**	**FDR**	**Rap1 -/- status**
GO:0004497	monooxygenase activity	0.00596968	DOWN
GO:0016705	oxidoreductase activity, acting on paired donors, with incorporation or reduction of molecular oxygen	0.00698089	DOWN
GO:0042802	identical protein binding	1.06E-02	UP
GO:0020037	heme binding	0.0163961	DOWN
GO:0016709	oxidoreductase activity, acting on paired donors, with incorporation or reduction of molecular oxygen, NADH or NADPH as one donor, and incorporation of one atom of oxygen	0.0163961	DOWN
GO:0005549	odorant binding	0.0163961	DOWN
GO:0005550	pheromone binding	0.0163961	DOWN
GO:0046906	tetrapyrrole binding	0.0170305	DOWN
