# Supplementary figures and images for: RAP1 Protects from Obesity through Its Extratelomeric Role Regulating Gene Expression
Source: Cell Rep. Author manuscript; Available in PMC 2018 Apr 7. (PMC5889507; doi:10.1016/j.celrep.2013.05.030)

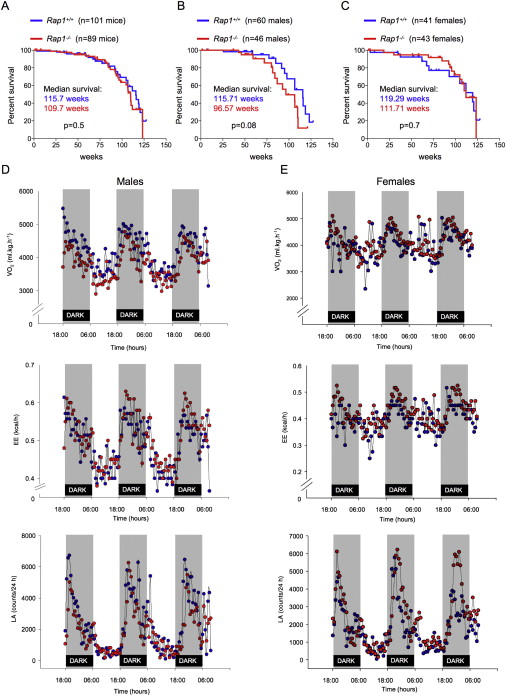

Supplement: Figure S1 [file NIHMS948947-supplement-Figure_S1.jpg]

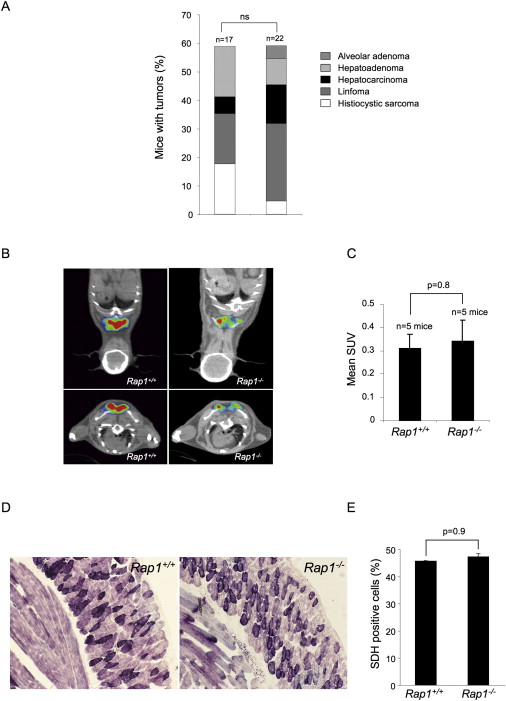

Supplement: Figure S2 [file NIHMS948947-supplement-Figure_S2.jpg]

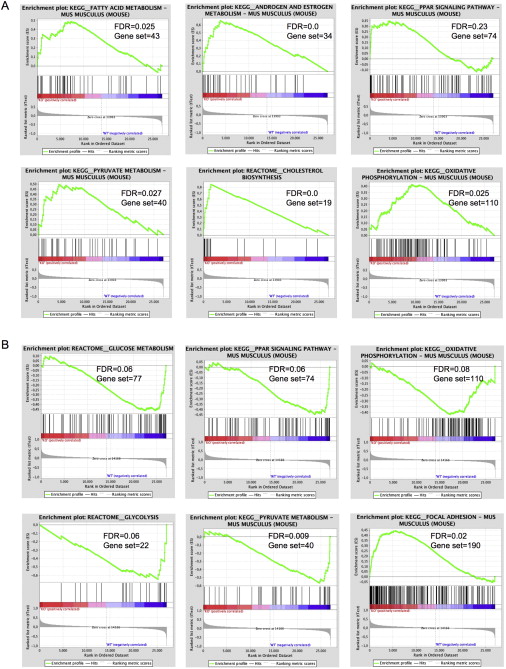

Supplement: Figure S3 [file NIHMS948947-supplement-Figure_S3.jpg]

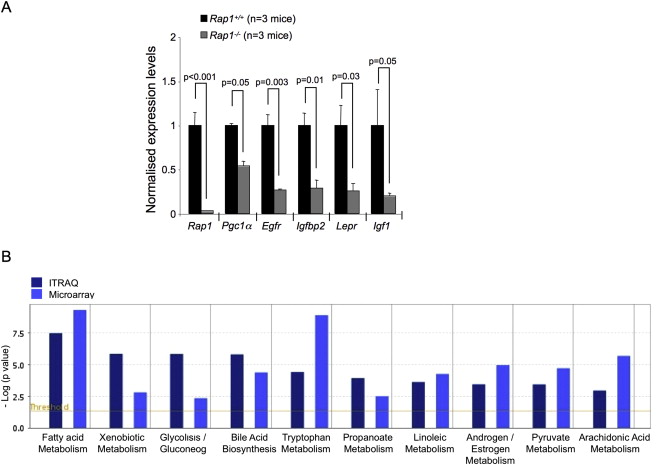

Supplement: Figure S4 [file NIHMS948947-supplement-Figure_S4.jpg]

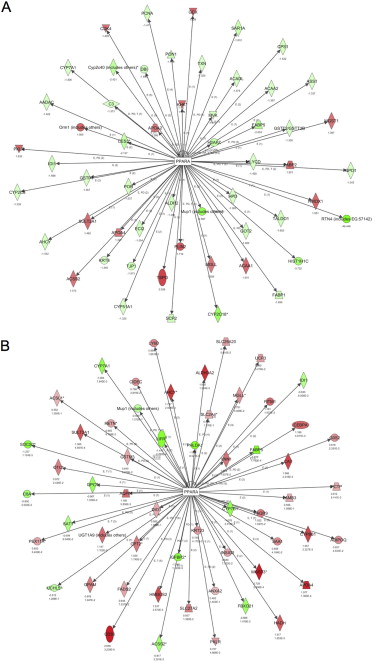

Supplement: Figure S5 [file NIHMS948947-supplement-Figure_S5.jpg]

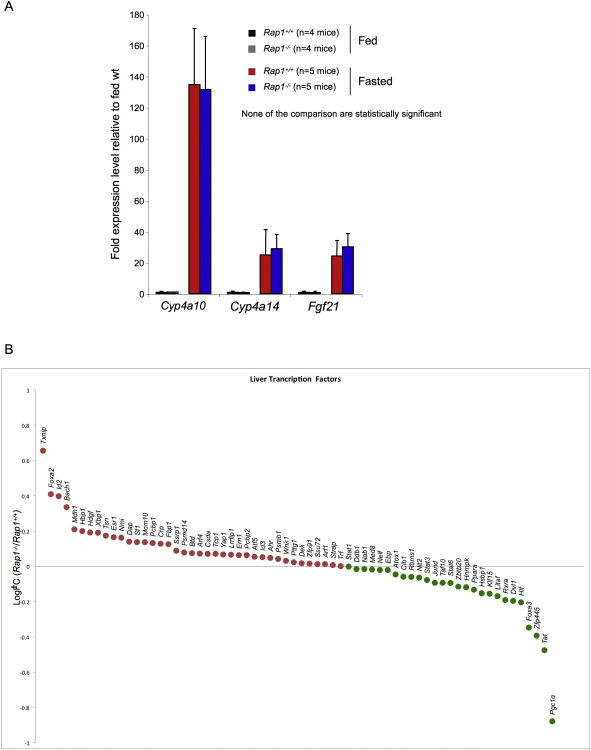

Supplement: Figure S6 [file NIHMS948947-supplement-Figure_S6.jpg]

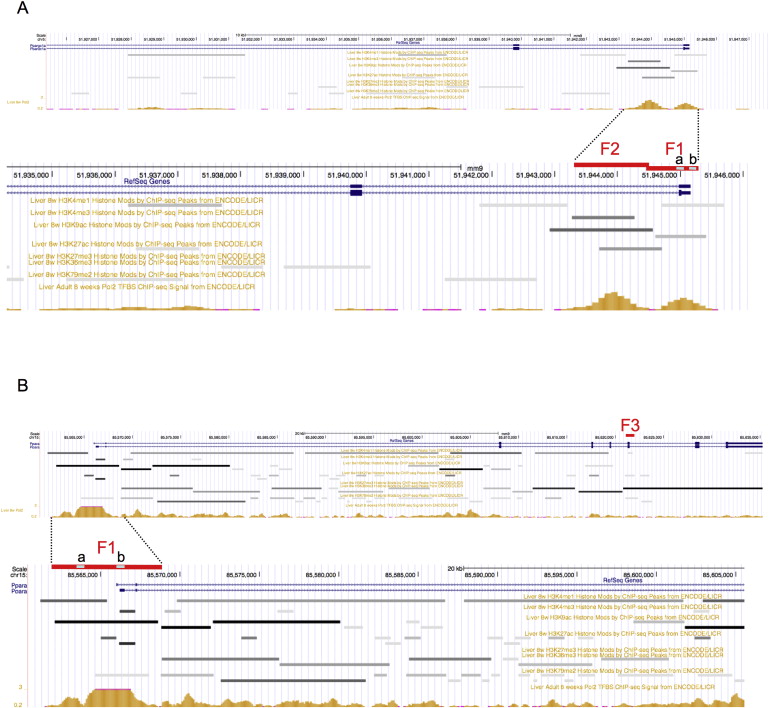

Supplement: Figure S7 [file NIHMS948947-supplement-Figure_S7.jpg]
